# An Iterative, Frequentist Approach for Latent Class Analysis to Evaluate Conditionally Dependent Diagnostic Tests

**DOI:** 10.3389/fvets.2021.588176

**Published:** 2021-02-10

**Authors:** Clara Schoneberg, Lothar Kreienbrock, Amely Campe

**Affiliations:** Department of Biometry, Epidemiology and Information Processing, WHO Collaborating Centre for Research and Training for Health in the Human-Animal-Environment Interface, University for Veterinary Medicine Hannover, Hannover, Germany

**Keywords:** conditional dependence, sensitivity, specificity, maximum likelihood, veterinary medicine

## Abstract

Latent class analysis is a well-established method in human and veterinary medicine for evaluating the accuracy of diagnostic tests without a gold standard. An important assumption of this procedure is the conditional independence of the tests. If tests with the same biological principle are used, this assumption is no longer met. Therefore, the model has to be adapted so that the dependencies between the tests can be considered. Our approach extends the traditional latent class model with a term for the conditional dependency of the tests. This extension increases the number of parameters to be estimated and leads to negative degrees of freedom of the model, meaning that not enough information is contained in the existing data to obtain a unique estimate. As a result, there is no clear solution. Hence, an iterative algorithm was developed to keep the number of parameters to be estimated small. Given adequate starting values, our approach first estimates the conditional dependencies and then regards the resulting values as fixed to recalculate the test accuracies and the prevalence with the same method used for independent tests. Subsequently, the new values of the test accuracy and prevalence are used to recalculate the terms for the conditional dependencies. These two steps are repeated until the model converges. We simulated five application scenarios based on diagnostic tests used in veterinary medicine. The results suggest that our method and the Bayesian approach produce similar precise results. However, while the presented approach is able to calculate more accurate results than the Bayesian approach if the test accuracies are initially misjudged, the estimates of the Bayesian method are more precise when incorrect dependencies are assumed. This finding shows that our approach is a useful addition to the existing Bayesian methods, while it has the advantage of allowing simpler and more objective estimations.

## Introduction

Information about the occurrence of livestock diseases in an animal population is important in many applications, such as surveillance and vaccination programs or the verification of the freedom from the disease. Therefore, the disease status of the individual animals is assessed using a diagnostic test. However, every test also has a number of incorrect results, which, depending on the disease, may have serious economic, social or political consequences. This result can be avoided by sequentially examining a subset of the animals with a different test or by testing all the animals with multiple diagnostic tests from the outset and thus confirming the diagnosis (1).

The latter was the chosen approach in a field trial to determine the prevalence of *Brucella abortus* in cattle in Northern Ireland in 2003/2004 (2). Each animal was examined with six serological tests simultaneously, which yielded conflicting results for a large number of the animals. For example, there were two positive and four negative test results for one animal. Knowledge of the test accuracy and therefore the probability of a false diagnosis of the tests are the basis for assessing the true underlying disease status. This information is obtained by evaluating the diagnostic tests used. To assess the sensitivity and specificity, the diagnostic test is usually compared with a gold standard test or is applied to animals with known disease status (1).

However, the test accuracy depends on many biological factors, such as the animal species, race, sex and immune history. For this reason, the diagnostic test accuracy varies across populations, and the values obtained in clinical evaluation studies are only conditionally applicable to the field settings (3). Therefore, if the exact values of the test accuracy for a set of diagnostic tests in a field study are of interest and a gold standard or animals with known disease status are not available, a latent class model can be used (4).

In this context, latent class analysis (LCA) is based on the assumption that observed categorical indicators imperfectly measure an underlying latent structure. By sampling the values of the categorical variable for a set of observations, the method is able to discover the latent structure and the error in the indicators. Applying this principle to the field of diagnostic test evaluation, the true unknown disease status is measured by observed diagnostic tests. By analyzing the response pattern of a set of tests, the prevalence of the disease in the sample and the diagnostic accuracy of every test used in this model can be discovered. Hence, the specific test performance under the given conditions such as the study settings and the structure of the subpopulation can be estimated.

The Bayesian approach to latent class models of the test accuracy is widely used in veterinary medicine (5). However, latent class analyses can also be implemented in a frequentist framework, and the model parameters can be estimated by classical inference (6, 7). This method does not require any prior distribution and therefore allows an easy and objective estimation of the parameters. A prior distribution can be a benefit for the estimation if there is much information that can be incorporated into the model. However, when new or less established tests are used, there is limited previous knowledge and it is difficult to determine an informative prior distribution. The Bayesian model requires also a burn-in phase and is often more time consuming, since the convergence and therefore the termination condition are difficult to assess. Consequently, it seems reasonable to use classic frequentist methods, especially when the user has little or no prior knowledge of the distributions due to the biological methods used. Regardless of the chosen method, the basis for most latent class models is that (i) the tested individuals can be divided into two or more populations with different prevalence values; (ii) the tests have the same sensitivity and specificity in all populations; and (iii) the tests are conditionally independent (i.e., given the true disease status). These assumptions are known as the Hui-Walter paradigm (6). However, in many cases, the assumption of conditional independence is hard to justify, especially if the tests are based on the same biological principle, such as the detection of antibodies. Ignoring the dependency structure among the diagnostic tests will introduce bias in the estimates so that a positive association will overestimate the test properties while a negative association will lead to an underestimation of the test accuracy (8).

Some Bayesian methods allow the consideration of conditionally dependent tests. A fixed effects model and a random effects model for data from a single population were developed in one approach (9), while another study used a simple method for two diagnostic tests that can be applied when two or more populations are studied (10). Moreover, three different models were described for varying forms of conditional dependence (11).

There are also some frequentist approaches for incorporating a dependence structure into the latent class analysis. A latent marginal model (12), different random effect models (13–15) and a more generalized mixture model (16) have been proposed. Another approach uses log-linear and logistic models to investigate conditional dependence (17). However, this model assumes that the true disease status of each individual is available, which is not possible in most diagnosis applications.

Although the solutions addressed above are available, we propose a frequentist method for estimating the prevalence, diagnostic test accuracy and dependence structure because we would like to present an easy-to-apply approach even for situations with no accessible prior information. The solution was intended to fit even when only three diagnostic tests are available and the status of each individual is unknown. We present the model as well as the algorithm and discuss its performance in different simulated scenarios, which were adopted from real-world examples in veterinary medicine to examine the performance of our method under different circumstances. The non-mathematically inclined reader may skip the following three subsections describing the statistical model.

## Materials and Methods

### The Latent Class Model

In a latent class model, it is assumed that there is a latent variable with *C* classes. The proportion of each class is estimated by *M* observed variables. Let the vector *Y*_*i*_ = (*Y*_*i*1_, …, *Y*_*iM*_), *i* = 1, …, *N* represent individual i's response pattern with the possible values 0, …, *r*_*m*_ for observation *Y*_*im*_. The probability of membership in the latent class *c* can be expressed as γ_*c*_ with $\overset{C-1}{\underset{c=0}{\sum }}\gamma_{c}=1$, and the probability of the response *r*_*m*_ to variable *m* in class *c* can be expressed as ρ_*m*,*r*_*m*_|*c*_. Let *I*(·) be the indicator function. Then, the likelihood of parameters γ and ρ for the observations *y* is denoted by Formula (a).

(a) $$\begin{array}{l} L\left(\gamma ,\rho |Y\;\right)=\overset{N}{\underset{i=1}{\sum }}\overset{C-1}{\underset{c=0}{\sum }}\gamma_{c}\overset{M}{\underset{m=1}{\prod }}\overset{R_{m}}{\underset{r_{m}=0}{\prod }}\rho_{m,r_{m}|c}^{I\left(y_{im}=r_{m}\right)}\; \end{array}$$

In this publication, we discuss the application to the diagnostic test evaluation for dichotomous diagnoses (i.e., with/affected vs. without/not affected). Thus, we only allow dichotomous responses for the observed variables and for two latent classes. This results in the simplified likelihood, which can be written as Formula (b).

(b) $$\begin{array}{l} L\left(\gamma ,\rho |Y\;\right)=\overset{N}{\underset{i=1}{\sum }}\overset{1}{\underset{c=0}{\sum }}\gamma_{c}\overset{M}{\underset{m\;=\;1}{\prod }}\overset{1}{\underset{r_{m}\;=\;0}{\prod }}\rho_{m,r_{m}|c}^{I\left(y_{im}\;=\;r_{m}\right)}\; \end{array}$$

It differs from the two-test, two-population scenario originally proposed by Hui and Walter (6) only in the number of populations and tests used. However, due to this parameter changes there are no closed-form maximum-likelihood solutions for this model and the values are estimated iteratively.

Key assumption of the approach is the conditional independence (i.e., given the true disease status) of all the tests (6). Therefore, within a latent class, the result of one test does not give any indication of the result of the other tests. If there are diagnostic tests with the same biological principle used in the analysis, that assumption no longer holds because the same external factors influence their diagnoses. As ignoring this underlying dependence structure leads to biased results (8), a term to describe the dependency must be included when using tests with the same (biological) testing principle.

### Conditional Dependence—The Interaction Term

If diagnostic tests are independent, the conditional response probabilities result from the product of the tests' individual response probabilities as described in the likelihood (b). It can be written in the simplified term (c) for three diagnostic tests *i, j*, and *k*.

(c) $$\begin{array}{l} \rho_{i,r_{i}|c}\cdot \rho_{j,r_{j}|c}\cdot \rho_{k,r_{k}|c}=P\left(r_{i},r_{j},r_{k}|c\right)\; \end{array}$$

However, if the tests are conditionally dependent on the underlying disease status, these dependencies must be taken into account when calculating the conditional item response probabilities. They are determined within the two latent classes containing the observations with a positive and a negative disease status, respectively.

The pairwise dependencies $\eta_{ij}^{c}$ of tests *i* and *j* conditional on the latent class *c* are estimated by comparing the observed matching correct response patterns *P*(*c, c*|*c*) with the test accuracies of the diagnostic tests used. If the observed test agreement is stronger than expected based on the known test accuracy, there is a positive dependency between the tests that cannot be attributed to the underlying disease status alone. If the test agreement is weaker than expected, there is a negative dependency. Formula (d) denotes the dependency of the diagnostic tests conditional on the latent class of the not affected/diseased animals *c* = 0, with *Sp*_*i*_ and *Sp*_*j*_ denoting the known specificities of tests *i* and *j*.

(d) $$\begin{array}{l} \eta_{ij}^{0}=P\left(0,0|0\right)-Sp_{i}Sp_{j}\; \end{array}$$

The dependency of three tests *i, j* and *k* can be estimated analogously by calculating the difference between their observed test agreement *P* (*c, c, c*|*c*) and the expected proportion of correct observations in class *c*. However, since the tests are pairwise dependent on each other, these values must be taken into account, as they are expected to influence the result. Due to their dependency, matching test results have an increased item response probability (i.e., prevalence) compared to the independent cases whereas the probabilities of the deviating test results decrease. This means that the pairwise dependencies must be added to the product of the test accuracies to calculate the expected test agreement. In addition, a possible dependency of the pairwise dependent tests on the third test is considered by multiplying the dependency term by the test accuracy of the third test. This results in the conditional dependency of three tests in the latent class of the not affected/diseased animals *c* = 0 to be calculated as described in Formula (e).

(e) $$\begin{array}{l} \eta_{ijk}^{0}=P\left(0,0,\;0|0\right)-\left(Sp_{i}Sp_{j}Sp_{k}+\eta_{ij}^{0}Sp_{k}+\eta_{ik}^{0}Sp_{j}+\eta_{jk}^{0}Sp_{i}\right)\; \end{array}$$

The conditional dependencies for the class of the in-/affected animals *c* = 1 can be calculated analogously.

Some authors use the terms “dependency” and “correlation” interchangeably (18, 19). We prefer “dependency” and therefore use it in this publication because the related term calculates concordance rather than a traditional correlation of quantitative variables.

The latent disease status determines the correct diagnosis of an observation, while other external factors trigger a misdiagnosis. Thus, only matching incorrect results are of interest to assess the dependency of the tests. The proportion of incorrect results, i.e., the proportion of incorrectly diagnosed animals, is determined by the accuracy of the test, which causes specific restrictions for the dependency parameter settings (20). This means, the higher the accuracies, the greater the agreement due to the correct diagnosis and the smaller the possible dependency. For example, if both tests have test accuracies of 100%, then all of their results match and their conditional dependency is zero. Therefore, the test accuracy of the examined tests determines the possible range of the dependencies.

For the dependencies to be comparable, they have to be detached from the test accuracies. In case of pairwise dependencies, this is achieved by standardizing Formula (d) to Formula (f). The three-test dependencies are standardized analogously.

(f) $$\begin{array}{l} Z_{ij}^{0}=\;\frac{\eta_{ij}^{0}}{\sqrt{Sp_{i}\left(1-Sp_{i}\right)}\sqrt{Sp_{j}\left(1-Sp_{j}\right)}}\; \end{array}$$

This model is applicable in situations when the results of at least two diagnostic tests are available (21). However, here, we focus on the three-test model.

The dependencies of the diagnostic tests are calculated within both latent classes and remain constant for all possible combinations of the results of the three tests in the respective class. Therefore, these values can also be determined by using all observed response patterns. Only the signs of the dependencies have to be adjusted due to matching or differing test results. Changing these equations results in the functions (g) of the conditional items response probabilities for the class of non-infected animals *c* = 0.

(g) $$\begin{array}{l} P\left(0,0,0|0\right)=Sp_{1}Sp_{2}Sp_{3}+\eta_{12}^{0}Sp_{3}+\eta_{13}^{0}Sp_{2}+\eta_{23}^{0}Sp_{1}+\eta_{123}^{0}\\ P\left(0,0,1|0\right)=Sp_{1}Sp_{2}\left(1-Sp_{3}\right)+\eta_{12}^{0}\left(1-Sp_{3}\right)-\eta_{13}^{0}Sp_{2}\\ -\eta_{23}^{0}Sp_{1}-\eta_{123}^{0}\\ P\left(0,1,0|0\right)=Sp_{1}\left(1-Sp_{2}\right)Sp_{3}-\eta_{12}^{0}Sp_{3}+\eta_{13}^{0}\left(1-Sp_{2}\right)\\ -\eta_{23}^{0}Sp_{1}-\eta_{123}^{0}\\ P\left(0,1,1|0\right)=Sp_{1}\left(1-Sp_{2}\right)\left(1-Sp_{3}\right)-\eta_{12}^{0}\left(1-Sp_{3}\right)\\ -\eta_{13}^{0}\left(1-Sp_{2}\right)+\eta_{23}^{0}Sp_{1}+\eta_{123}^{0}\\ P\left(1,0,0|0\right)=\left(1-Sp_{1}\right)Sp_{2}Sp_{3}-\eta_{12}^{0}Sp_{3}-\eta_{13}^{0}Sp_{2}\\ +\eta_{23}^{0}\left(1-Sp_{1}\right)-\eta_{123}^{0}\\ P\left(1,0,1|0\right)=\left(1-Sp_{1}\right)Sp_{2}\left(1-Sp_{3}\right)-\eta_{12}^{0}\left(1-Sp_{3}\right)+\eta_{13}^{0}Sp_{2}\\ -\eta_{23}^{0}\left(1-Sp_{1}\right)+\eta_{123}^{0}\\ P\left(1,1,0|0\right)=\left(1-Sp_{1}\right)\left(1-Sp_{2}\right)Sp_{3}+\eta_{12}^{0}Sp_{3}-\eta_{13}^{0}\left(1-Sp_{2}\right)\\ -\eta_{23}^{0}\left(1-Sp_{1}\right)+\eta_{123}^{0}\\ P\left(1,1,1|0\right)=\left(1-Sp_{1}\right)\left(1-Sp_{2}\right)\left(1-Sp_{3}\right)+\eta_{12}^{0}\left(1-Sp_{3}\right)\\ +\eta_{13}^{0}\left(1-Sp_{2}\right)-\eta_{23}^{0}\left(1-Sp_{1}\right)-\eta_{123}^{0} \end{array}$$

The item-response probabilities conditional on the latent class of the affected/diseased animals *c* = 1 are determined analogously.

The (standardized) dependency (e) indicates the strength of the interdependence of the tests, i.e., the share of their concordant false diagnoses. To interpret this measure, the size and direction should be considered. If two tests completely agree on their incorrect diagnosis (i.e., both tests assign the incorrect disease status to exactly the same animals), then they have a (standardized) dependency of 1. If the two tests agree only at random regarding the incorrect diagnosis of the disease status, the observed agreement between these tests matches the expected agreement between two independent tests. Hence, the (standardized) dependency is zero. The dependency is negative when there are fewer matching results than expected by chance. Thus, this measure has an interpretation similar to Cohen's Kappa. However, here, negative values play a subordinate role in the application to diagnostic tests, since similar testing principles tend to lead to increased agreement in incorrect decisions. It is very unlikely that two tests with a similar test procedure have a negative dependency, as this phenomenon would imply that higher biological similarity leads to a lower level of agreement.

### Conditional Dependence—Illustrative Examples

To obtain a better idea of the magnitude of the conditional dependency, published studies that contain observations with a confirmed latent status may be discussed. Although publications with the information needed are rare and provide a rather rough indication on the size of the dependencies realized under the given study conditions, they may yield a valuable starting value for subsequent analyses. The following examples set the framework for the magnitude of the dependency in our simulation.

First, we calculated the standardized dependency from study data on toxoplasmosis in pigs (10). In that study, the dependency between a microscopic agglutination test and an ELISA was 0.33 for the positive results and 0.49 for the negative results. In another investigation, the direct detection of *Strongyloides* infection in the stools of Cambodian refugees in Canada was compared with a serological examination (22). Based on these data, we calculated dependencies of 0.18 and 0.17 for the positive and negative observations, respectively.

### The Algorithm

In the last subsections it was described how the classical frequentist latent class analysis can be extended by a term to describe the conditional dependencies between the diagnostic tests. Due to the general misspecification of the describing parameters within the setting of conditional dependency, an iterative algorithm is proposed. Here, we present a solution for the use of three diagnostic tests. The basic idea of the algorithm is to consider alternately the test accuracies and the conditional dependencies as fixed values. Thus, the method presented here has the advantage of always resulting in a positive number of degrees of freedom in each iteration step for three tests in one population compared to other methods for the estimation of test accuracies for conditionally dependent tests [e.g., (10, 12, 13, 21)]. The algorithm includes the following steps:

- Choose suitable starting values for the test accuracies and the conditional dependencies between the tests.
- Consider the conditional dependencies as fixed. Execute the expectation maximization (EM) algorithm to estimate the best-fit test accuracies for the data. For this step, we followed the EM algorithm described in a conditional independent latent class approach (7) and slightly adopted it by replacing the conditional item response probabilities described in Formula (b) by the conditionally dependent item response probabilities (g) in the likelihood function.
- Recalculate the conditional dependency in two substeps:
  1. Use the conditional dependencies and the test accuracies from the previous step to calculate the latent class membership probabilities *P*(*r*_1_, *r*_2_, *r*_3_|*c*) of the observations by using Formula (g). An observation is assigned to the class for which it has the highest probability.
  2. With the knowledge of the latent status, determine the conditional dependency by using Formulas (d, e).
- Start again with step (ii) until the model converges, i.e., the log-likelihood of two consecutive models differs by <0.00001 or the algorithm reaches 1,000 iterations.

We implemented the algorithm in R [version 3.5.0; (23)] by programming three main functions. The first function calculates the dependencies for fixed test accuracies and a set of observed response patterns, the second one determines the test accuracies and the prevalence for the given dependencies and response patterns (EM algorithm) and the third one combines the first two functions by calling them alternately (see Supplementary File 27 for complete source code).

### Simulation Study—General Framework

We tested the applicability of the algorithm for three diagnostic tests in a single population by conducting a simulation study. Therefore, we took different combinations of the test accuracies, prevalence and conditional dependencies into account. These scenarios allowed an assessment of the performance of the iterative approach presented in this publication compared with that of the conditionally independent latent class analysis and the Bayesian approach for conditionally dependent tests. All the simulation scenarios are motivated by diagnostic tests used in veterinary medicine. As a small sample size leads to an increased margin of error, we simulated 10,000 observations.

We considered the following cases:

(1) Three independent diagnostic tests with high test accuracies in a population with a moderate prevalence:

Diagnostic tests are conditionally independent if they are based on different biological principles, for example, if a tumor is detected using physical examination, medical imaging (e.g., sonography) and microscopic examination of a tissue sample. Scenarios such as this one should not cause any problems in the conditionally independent latent class analysis and result in very accurate estimates of that method. Therefore, it should provide the same results in the new approach presented in this publication as well as in models assuming conditional independent diagnostic tests. Hence, this scenario serves as basic validation for the newly fitted model.

(2) Two highly dependent tests with low test accuracies and a third test with low dependencies and high test accuracies in a population with a high prevalence:

This scenario may be the most problematic in the conditionally independent latent class model: The two dependent tests may cause many matching results that lead to an overestimation of their test accuracies and underestimated values in the third test. This situation applies, for instance, to the diagnosis of infectious diseases with one antibody test applied to two different sample types (e.g., serum, feces, milk, etc.) and the often more accurate direct detection of the pathogen (24). By using the same test twice, the results are highly interdependent. The third test uses a different detection method and is therefore independent of the other results. This scenario may act as validation for the method proposed here to identify dependency structures in data and improve the correctness of model estimates.

(3) Two highly dependent tests with low test accuracies and a third test with low dependencies and high test accuracies in a population with a low prevalence:

This is generally the same scenario as (2) but with a lower prevalence, which is common for many diseases. In this case, only a small proportion of the sample contains positive responses compared to the other possible response patterns. This phenomenon makes the estimation of both the prevalence and sensitivities more ambiguous and therefore more prone to errors.

(4) Three diagnostic tests with moderate test accuracies and medium dependencies in a population with a high prevalence:

In this scenario, all three tests are conditionally dependent on each other, resulting in an overestimation of their test accuracies in the latent class model, which assumes conditional independence. For instance, three different veterinarians may perform a physical examination on the same group of animals with a suspected disease. They all have different qualifications and therefore different diagnostic sensitivities and specificities. However, experience in the same work environment with the same time and budgetary constraints can be the cause of consistent misdiagnoses (25). This scenario serves as validation for the method proposed to recognize dependency structures and calculate more accurate values.

(5) Three diagnostic tests and a population with values for the test accuracies and the prevalence from a practical example with estimated values for the dependency structure:

To ensure that the procedure is evaluated under realistic conditions, this scenario uses the results from a prevalence study for *Brucella abortus* in cattle in Northern Ireland (2) that was mentioned in the introduction. It simulates a population at risk with a high prevalence. In the study, six different antibody tests were used from which we selected three for the simulation: an indirect ELISA, a competitive ELISA and a serum agglutination test. Since we had no information about the exact test dependencies of these tests, we based the simulations on their accuracies and biological principles. The two ELISA tests have low sensitivities, so they have a high proportion of false negative results, which may have similar causes in many cases due to similar biological principles and results in a high level of dependency in the positive latent class. As both tests have high specificities, similar detection methods have only a minor influence on the dependencies of the negative latent class. The serum agglutination test uses a different approach, but it is also based on the detection of antibodies; therefore, we assume it is only slightly dependent on the other tests.

Table 1 shows the parameter settings in the five simulated scenarios in detail. We considered only positive pairwise dependencies since negative values are not biologically justifiable.

**Table 1:** Input parameter values for the data simulation of the five scenarios.

**Scenario**	**Scenario 1**	**Scenario 2**	**Scenario 3**	**Scenario 4**	**Scenario 5**
Prevalence in %	30.00	40.00	3.00	40.00	20.00
Sensitivity Test 1	90.00	90.00	90.00	80.00	72.00
Sensitivity Test 2	85.00	70.00	70.00	66.00	65.00
Sensitivity Test 3	90.00	65.00	65.00	70.00	97.00
Specifity Test 1	95.00	99.00	99.00	95.00	98.00
Specifity Test 2	95.00	80.00	80.00	85.00	99.00
Specifity Test 3	99.00	85.00	85.00	88.00	98.00
$\eta_{12}^{+}$ (Standardized)	0.000 (0.000)	0.000 (0.000)	0.000 (0.000)	0.038 (0.200)	0.129 (0.600)
$\eta_{13}^{+}$ (Standardized)	0.000 (0.000)	0.000 (0.000)	0.000 (0.000)	0.046 (0.250)	0.008 (0.100)
$\eta_{23}^{+}$ (Standardized)	0.000 (0.000)	0.121 (0.600)	0.121 (0.600)	0.087 (0.400)	0.012 (0.150)
$\eta_{123}^{+}$ (Standardized)	0.000 (0.000)	0.000 (0.000)	0.000 (0.000)	−0.004 (−0.050)	0.000 (0.000)
$\eta_{12}^{-}$ (Standardized)	0.000 (0.000)	0.000 (0.000)	0.000 (0.000)	0.016 (0.200)	0.001 (0.100)
$\eta_{13}^{-}$ (Standardized)	0.000 (0.000)	0.000 (0.000)	0.000 (0.000)	0.018 (0.250)	0.003 (0.150)
$\eta_{23}^{-}$ (Standardized)	0.000 (0.000)	0.086 (0.600)	0.086 (0.600)	0.046 (0.400)	0.001 (0.100)
$\eta_{123}^{-}$ (Standardized)	0.000 (0.000)	0.000 (0.000)	0.000 (0.000)	−0.001 (−0.050)	0.000 (0.000)

### Simulation Study—Starting Values

We applied nine different sets of starting values (6 well-chosen and 3 poorly chosen) to each of the five scenarios. As it can be assumed that prior knowledge of the applied tests, their dependency structure and the studied population is available, we considered six different sets of well-chosen “informative” starting values as follows (see Supplementary Table 1):

1. The correct values for the test accuracies, the dependency structure and the prevalence.
2. The correct values for the test accuracies and the prevalence; the dependency of the tests is stronger than that simulated.
3. The correct values for the test accuracies and the prevalence; the dependency of the tests is weaker than that simulated. The only exception is scenario 1 (independent tests): As weakening the dependency of independent tests leads to negative dependencies and negative dependencies are not biologically justifiable, another set of positive dependencies is used instead.
4. The correct values for the dependency structure; the test accuracies are better than those simulated, and the prevalence differs from the simulated value.
5. The correct values for the dependency structure; the test accuracies are poorer than those simulated, and the prevalence differs from the simulated value.
6. The values for the test accuracies, the dependency structure and the prevalence all differ (slightly) from the simulated data.

On the other hand, if a new diagnostic test is used, false assumptions about the underlying dependency structure, the test accuracy and even the prevalence are possible. Therefore, we also took three sets of poorly chosen starting values into consideration, that deviate greatly from the simulated values in terms of the test accuracy and the dependency structure (see Supplementary Table 2):

1. A value of 50% for all the test accuracies and the prevalence; the tests are assumed to be independent of each other.
2. Incorrect assumptions about which tests are dependent on each other, an incorrect ratio of the test accuracies and a prevalence that differs from the simulated value.
3. The results from the conditionally independent latent class analysis for the test accuracies and the prevalence as well as the incorrect dependencies.

### Simulation Study—Parameter Restrictions

In some cases, there are justifiable restrictions for the resulting parameter values. As an example, negative dependencies between two diagnostic tests with the same biological testing principle are very unlikely. Another example for a justifiable restriction is to set the dependencies of known independent tests fixed to zero. Since the test accuracies are already limited to the unit interval by the EM algorithm, further restrictions always depend on the situation and are therefore difficult to determine.

We examined the effect of parameter restrictions on the estimations of the iterative approach by repeating the calculations and adding restriction rules. As all limitations of the resulting parameter values require knowledge of the population, the disease and the diagnostic tests used in the study, they depend on the setting and are not generalizable. Thus, we focused on the most basic limitations and excluded unrealistic dependencies [standardized values < −1 or >1; (20)] and allowed only positive pairwise dependencies. Therefore, we modified the algorithm by adding an additional query to check whether the dependencies are within the newly defined limits. If that was not the case, they were adjusted in the next step. For this analysis, we limited ourselves to the scenario with the worst results (largest deviations from the simulated values) since these estimates needed the most improvement.

### Bayesian Estimation

We also calculated the results for the five scenarios (Table 1) in the Bayesian framework of the presented latent class model (see Supplementary File 28 for complete source code). For this, Formula (g) was implemented in the open source software OpenBUGS. In contrast to the presented iterative method, the algorithm requires prior information in the form of distribution functions. We used the package *rjags* in R (26) to calculate these priors. The basis for the calculation were the same starting values as in the iterative approach (Supplementary Tables 1, 2), but we also took a maximum possible uncertainty in the initial estimate of ±10% into account. OpenBUGS estimated the parameters using a Gibbs sampler, a special case of the Markov chain Monte Carlo (MCMC) algorithm (27). We ran the model with 10,000 iterations and a burn-in phase of 1,000 steps and compared the results with those of the iterative approach.

## Results

The five simulation scenarios consider different possible applications. Hence, we reflect on their results individually before we analyze them jointly to investigate possible differences. All results are shown in detail in Supplementary Tables 3–26.

### Scenario 1: Three Independent Tests (Supplementary Tables 3, 8, 15, 20)

All three latent class analysis approaches were able to estimate the parameters precisely when independence was initially assumed (see Supplementary Tables 3, 15). However, under the assumption of dependent tests with incorrect accuracies the results deviated up to 4% from the simulated values in the iterative, frequentist approach. In the Bayesian model, these deviations increased to up to 7%. Only the first set of poorly chosen starting values resulted in incorrect estimates of the prevalence (see Supplementary Table 8) in the iterative, frequentist approach.

Nevertheless, overall, the results showed that all three approaches are equally applicable for evaluating independent diagnostic tests.

### Scenario 2: Two Highly Dependent Tests in a Population With a High Prevalence (Supplementary Tables 4, 9, 16, 21)

The conditionally independent latent class analysis was not able to detect the connection between the tests (for none of the applied starting values) and therefore misjudged their accuracy by up to 20%. In contrast, the iterative method was able to determine the simulated parameters with only minor deviations of at most 8% for all well-chosen starting values (see Supplementary Table 4). However, the method yielded incorrect results for the accuracy parameters if the iterative process started with poorly chosen values (see Supplementary Table 9). This phenomenon was more prominent the more the ratio of the test accuracies and the dependencies differed from the simulated values. The Bayesian approach led to very similar results (see Supplementary Tables 16, 21). Thus, the algorithm is able to determine the correct parameter values, but initial information about the dependencies and the test accuracies is needed to choose the appropriate starting values.

### Scenario 3: Two Highly Dependent Tests in a Population With a Low Prevalence (Supplementary Tables 5, 10, 17, 22)

The low prevalence in this scenario complicated the estimation. While the conditionally independent latent class analysis resulted in strongly deviating and unrealistic values for the outcome (e.g., a sensitivity of 13% for test 1), the iterative approach was mostly able to calculate the simulated values by using well-fitting starting values. Only the results for the sensitivity of test 1 posed a problem in two cases with deviations of more than 50% (see Supplementary Table 5). The estimations of the specificities were more accurate.

**Figure 1:**
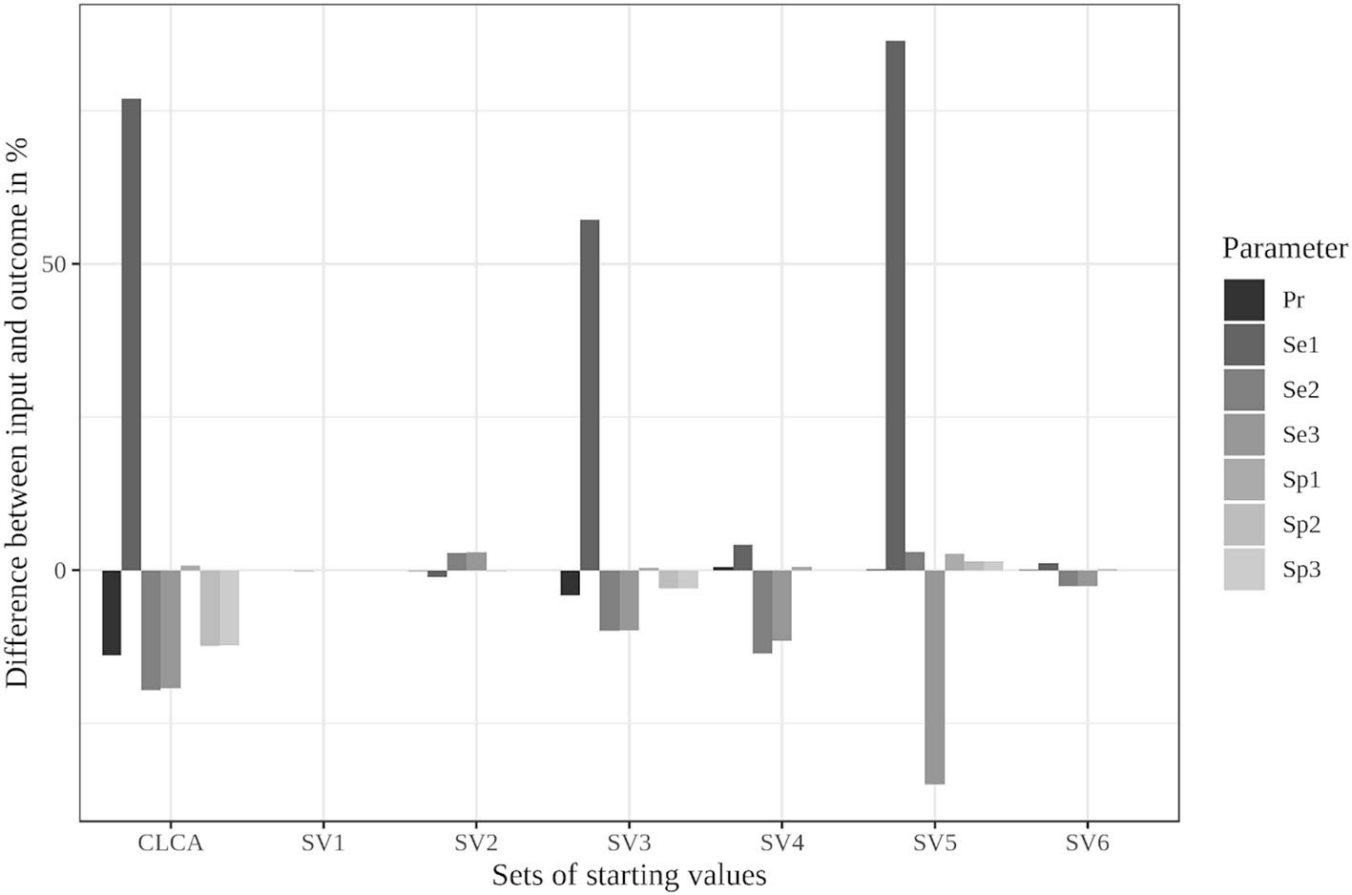
Differences between the configured input data and model outcomes using six sets of starting values for the latent class model assuming conditional independence and the iterative model under the conditions of scenario 3 [CLCA, conditionally independent LCA; SV1, set of starting values 1 (Sv2-Sv6 are defined analogously); Pr, Prevalence; Se1, Sensitivity of test 1; Sp1, Specificity of test 1 (Se2, Se3, Sp2, and Sp3 are defined analogously)].

This scenario resulted in the largest differences from the simulated values in the iterative approach (see Figure 1); therefore, we considered it for the modified algorithm with parameter restrictions.

The Bayesian approach led to better results than the frequentist method, as the deviations in the values for the sensitivity of test 1 reached a maximum of 11% for the well-chosen starting values (see Supplementary Table 17). In contrast, the divergences of the estimated specificity and prevalence were approximately the same size and therefore slightly worse than those from the frequentist approach. Poorly chosen starting values resulted in both methods in incorrect values (see Supplementary Tables 10, 22).

### Scenario 4: Three Moderately Dependent Tests With a High Prevalence (Supplementary Tables 6, 11, 18, 23)

The estimates of the conditionally independent latent class analysis deviated by up to 16% from the simulated values while the iterative method and the Bayesian approach were able to obtain results that were more precise (maximum deviations of 6 or 12%; see Supplementary Tables 6, 18).

However, there was one exception in the Bayesian approach. Starting value set 5 (underestimated prevalence and test accuracy) led to values that deviated up to 20% from the simulated values. The initial misjudgment of the test accuracies and the prevalence therefore has a stronger effect on the Bayesian approach in this scenario than on the frequentist method.

The results of the poorly chosen starting values differed considerably from the simulated values in both methods (see Supplementary Tables 11, 23).

### Scenario 5: *Brucellosis* Example (Supplementary Tables 7, 12, 19, 24)

While the conditionally independent latent class analysis overestimated the values by almost 20% in this simulation, all six sets of well-chosen starting parameters resulted in approximately correct values for the iterative approach. The deviations in the estimated sensitivities reached up to 8%, whereas they were at most 2% for the specificities and the prevalence (see Supplementary Table 7).

The Bayesian method resulted in similar values for most of the well-chosen starting values with deviations up to 6% for the sensitivities and 5% for the specificities and the prevalence (see Supplementary Table 19). However, the model did not converge using starting value set 3, and the algorithm aborted the calculation.

Both the iterative, frequentist approach and the Bayesian approach resulted in strongly deviating parameter values with the poorly chosen starting values (see Supplementary Tables 12, 24).

### Parameter Restrictions (Supplementary Tables 13, 14, 25, 26)

**Figure 2:**
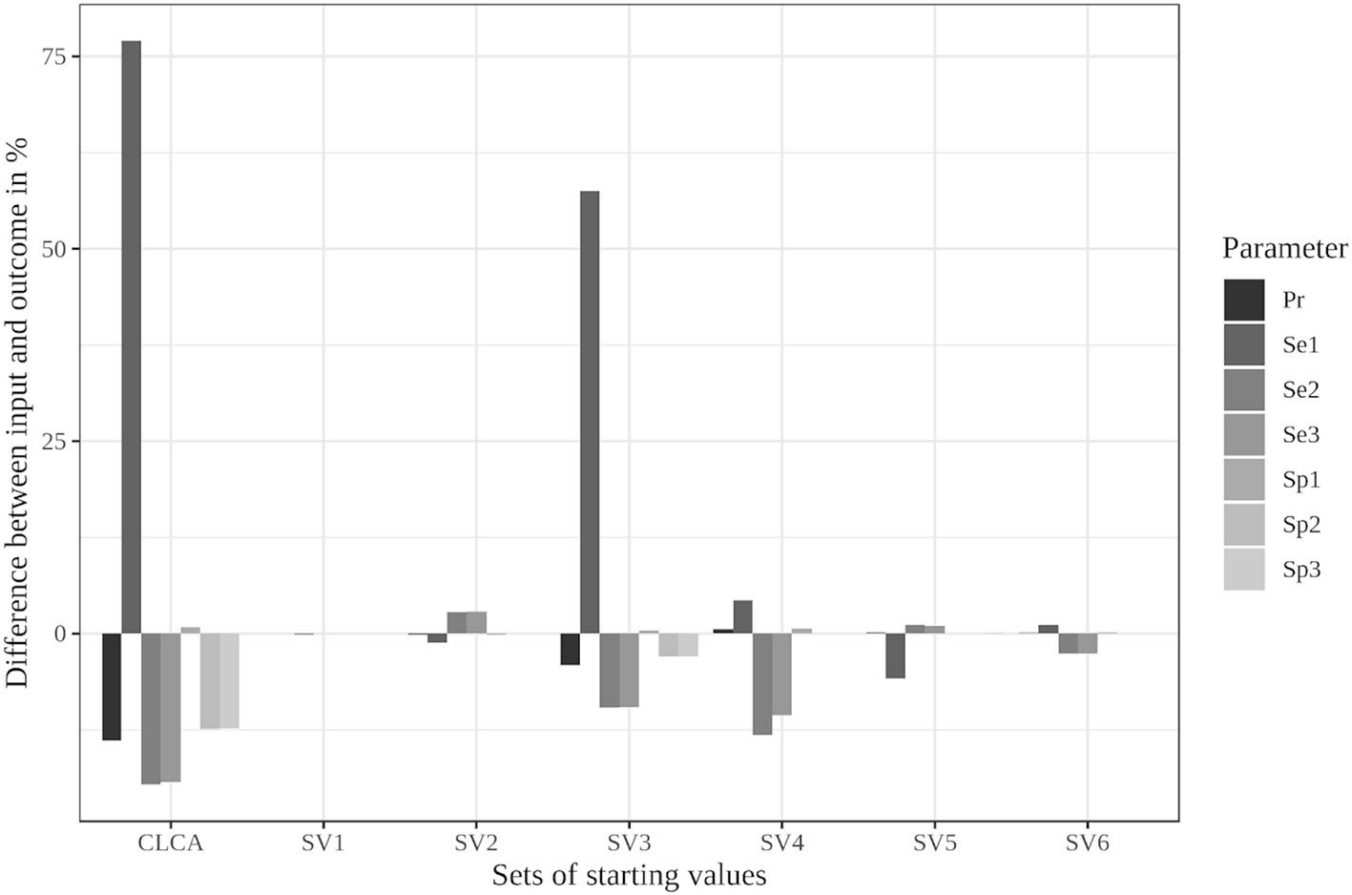
Differences between the configured input data and model outcomes using the six sets of starting values for the latent class model assuming conditional independence and by using the iterative model under the conditions of scenario 3 with only positive pairwise dependencies allowed [CLCA, conditionally independent LCA; SV1, starting values 1 (Sv2-Sv6 are defined analogously); Pr, Prevalence; Se1, Sensitivity of test 1; Sp1, Specificity of test 1 (Se2, Se3, Sp2, and Sp3 are defined analogously)].

The limitation to reasonable positive dependencies had no effect on the results for most starting values since the resulting dependencies were already within the defined boundaries. For starting value set 5, however, the differences between the input data and the outcome decreased remarkably (see Supplementary Table 13 and Figure 2). While it led to the strongest deviations in the unrestricted model, the restricted model was able to reduce the differences to a maximum of 6%. Even the poorly chosen starting values reached partially better results when the boundaries were considered (see Supplementary Table 14).

In contrast, the parameter restrictions had very little effect in the Bayesian model (see Supplementary Tables 25, 26). Most results did not change at all. Some values, especially the prevalence estimates, were slightly worse (0.5%) in the restricted model.

### Overall Results

The latent class analysis considering the dependency structure was able to calculate less biased parameter values than the classical frequentist latent class analysis for most of the informative stating values. Both the Bayesian and iterative methods produced very similar results (see Table 2). The estimated values differed considerably from the simulation settings if the prevalence of the simulated disease was low or if the starting values differed substantially from the simulated data. However, there were differences between the two methods. While the proposed iterative approach needed relatively accurate prior knowledge of the dependency structure, it was better able to deal with a slight-to-moderate initial misjudgment of the test accuracies and the prevalence than the Bayesian approach. However, in some rare situations, both methods are prone to restricted convergence or strongly biased results.

**Table 2:** Maximum deviations of the three compared methods to the simulated values in the five simulated scenarios with well-chosen starting values displayed as values in percent.

	**Independent LCA**	**Bayesian LCA**	**Iterative LCA**
Scenario 1	1.0	6.9	4.4
Scenario 2	20.4	6.9	7.7
Scenario 3	77.0	15.0	86.4^1^
Scenario 4	15.7	18.7^2^	5.9
Scenario 5	19.6	5.9^3^	8.1
Parameter restriction	77.0	15.1	57.5^1^

1 *Strongest deviation for the estimated sensitivity of test 1, the other parameters in the other scenarios had a maximum deviation of about 13%*.

2 *Strongest deviation for the estimations of starting values 5, the parameters in the other scenarios had a maximum deviation of 11.8%*.

3 *The model did not converge using starting values 3 and the algorithm aborted the calculation*.

Although the iterative approach yielded varying results for the different starting value sets, the associated log-likelihood always had the same value within all five simulation scenarios. This finding indicates that all results within a scenario, although they have very different values, represent a local maximum and are therefore equally likely under the observed responses. These results were estimated in only a few iteration steps in all five scenarios (a maximum of 9 iteration steps in scenario 3, see Supplementary Tables 5, 10). Thus, the convergence of the iterative approach was not noticeably slower than that of the conditionally independent latent class model. However, it was significantly faster than the convergence of the Bayesian method, for which 11,000 iterations were set.

## Discussion

In this publication, we presented an iterative, frequentist latent class approach for the evaluation of conditionally dependent diagnostic tests. We compared it to the Bayesian method and the classical conditionally independent analysis by performing a simulation study.

If two diagnostic tests with the same biological principle are used, the same reasons (e.g., cross-reactions, pathogen concentration) will lead to incorrect diagnoses, which strongly connect the outcome of these tests (28). Applying the classical latent class model without consideration of these dependencies leads to results that differ strongly from the true underlying parameters. The simulation studies presented in this publication confirmed this hypothesis.

These differences decrease or are even eliminated by using a model that considers the conditional dependencies. However, the accuracy of the estimates from the presented iterative method as well as the Bayesian approach strongly depends on two factors: the starting values and the size of the underlying parameters.

Starting values for the test accuracy of the diagnostic tests used can be obtained from the manufacturer's evaluation studies or from previous studies employing these tests. The conditional dependencies can be estimated by examining the biological methods of the tests and comparing them to each other. Similar procedures are more likely to be highly dependent (21). However, if there is no prior knowledge for the applied tests and no information on the biological procedures used, it is difficult to determine the correct starting parameters, and they must be chosen randomly. Thus, there is no assurance that the selected parameters are actually correct. However, if good estimates are available, the presented iterative procedure will be able to find the right parameters in just a few steps.

The size of the underlying parameters also influences the quality of the estimates. For scenario 3 with a low prevalence and a strong dependency, all the compared methods attained suboptimal performance within the simulation study. This deteriorated accuracy in populations with low prevalence was also observed in the Bayesian framework in simulation studies (29). This finding could cause problems in applications since there are many diseases with low prevalence in veterinary medicine [e.g., (30, 31)]. However, there were differences in the results of the two approaches due to the different estimation methods. The iterative approach estimated the specificities of the presented simulation scenarios, especially in the third scenario, more precisely than the Bayesian method. Due to the higher values of the specificity, many consistently negative test results were correctly assigned to the negative latent class in the first step of the algorithm regardless of the starting values. The incorrectly assigned positive observations had little influence due to the low prevalence. Thus, higher test accuracies lead to a clearer separation of the latent classes and a greater tolerance toward poorly chosen starting values. Furthermore, the more the starting values deviated from the given settings, the greater the deviations in the results were. While the presented iterative approach fared slightly better with an initial misjudgment of the test accuracies and the prevalence, the Bayesian method led to a more accurate convergence when incorrect dependencies were initially assumed. Therefore, the best latent class method for conditionally dependent diagnostic tests depends on the study population, the tests used and the accuracy of the existing prior knowledge.

Despite this phenomenon, the log-likelihoods of the different results within each scenario, the correct and the incorrect ones, converge to the same value. This finding suggests that the function has several maxima. Each result found by our method represents one local maximum and all these maxima are equally probable with the given dataset. Therefore, this model is not able to find a unique solution and well-chosen starting values are needed to ensure convergence to the correct parameter values. The reason is that the addition of dependency terms increases the number of parameters to be estimated, while the information provided by the observed response pattern does not change. The proposed method takes this property into account by estimating parameters in a stepwise algorithm that regards the dependency terms and the test accuracies alternately as fixed. As a result, there is a positive number of degrees of freedom in each step of the algorithm, and the identifiability is improved. The model is applicable to situations in which results from at least two diagnostic tests are available. However, as the two-test case was already underidentified without the additional dependency terms (df = −1) and therefore had no chance for a unique solution in the proposed iterative approach, we focused our analyses on the special case of three diagnostic tests. However, the increased number of tests is not sufficient for a clear result.

The ambiguity of the solutions occurs regardless of the chosen method, as the addition of dependency terms results in more parameters to be estimated than information is available in the data. Therefore, there is no model that takes the dependency between all tests into account and comes to a unique solution. This lack of knowledge has to be replaced by accurate prior information. If the priors are (unknowingly) false, the model is not always able to find the right solution for some parameter compositions. This limitation causes uncertainty regarding the assumptions at the beginning of the analysis; i.e., the results are not reliable, and not even an iterative calculation is able to solve this problem. Thus, good prior knowledge is necessary for accurate estimates, and uninformative priors should not be used with this method (the Bayesian or the iterative, frequentist approach). Other researchers have already observed and pointed out the importance of justified priors in the Bayesian framework (32, 33). Nevertheless, the Bayesian approach is able to express the certainty in the prior knowledge in the form of distribution functions, which may help to reduce the impact of initial misjudgments but also makes the modeling more complex, while the iterative, frequentist approach may include more “practical-use knowledge,” i.e., information on the general biological framework of the diagnostic method used.

Establishing boundaries for the dependencies improves the parameter estimates from the iterative approach further if the dependencies are within a certain interval and the values outside the interval can be excluded with certainty. These boundaries can prevent major deviations in the results, as shown in the second calculation of scenario 3. However, parameter restrictions should be used with caution. Only unrealistic values for a certain application should actually be excluded. If that is not the case, a true underlying parameter value may unknowingly be rejected as a possible solution, and the algorithm is no longer able to calculate the correct parameter set. Hence, the restrictions help to improve parameter estimation but also bear the risk of excluding the correct results from the start by choosing incorrect limits. Thus, if one is unsure of which limits to choose, it is better to completely remove them and carry out the estimation only with the best possible starting values.

Overall, the fit of the latent class model and the parameter estimates can be improved by allowing an interaction term. If the results of three diagnostic tests are available, both the Bayesian method and the iterative, frequentist approach presented in this paper are strongly dependent on the prior information due to the lack of information in the data. If there is insufficient knowledge about the test accuracies, the prevalence and the dependencies of the tests, and hence, these values are initially misjudged, both methods will lead to incorrect results. Extensive prior knowledge is therefore the basis for the applicability of the latent class analysis considering of conditional dependencies, both in the Bayesian and frequentist frameworks.

## Conclusion

The presented simulation study showed that considering a possible dependency structure improves the estimation in a latent class analysis. However, it was unable to clearly determine which method resulted in more accurate values overall, as the iterative, frequentist approach and the Bayesian approach performed differently in the presented scenarios. While both methods are dependent on prior knowledge in the form of well-chosen starting values and prior distributions, the simulation studies carried out in this publication suggest that the iterative, frequentist method requires previous knowledge that is oriented more toward practical experience and therefore may be easier to obtain.

Overall, the simulation studies presented here indicate that the iterative, frequentist approach is an appropriate method to evaluate conditionally dependent diagnostic tests.

## Data Availability Statement

The original contributions presented in the study are included in the article/Supplementary Material, further inquiries can be directed to the corresponding author/s.

## Author Contributions

CS and AC: conceptualization. CS: data curation, formal analysis, investigation, methodology, software, validation, and writing—original draft. LK and AC: project administration and supervision. CS, LK, and AC: writing—review and editing. All authors contributed to the article and approved the submitted version.

## Conflict of Interest

The authors declare that the research was conducted in the absence of any commercial or financial relationships that could be construed as a potential conflict of interest.
