# Supplementary material for: An Iterative, Frequentist Approach for Latent Class Analysis to Evaluate Conditionally Dependent Diagnostic Tests
Source: Front Vet Sci. 2021 Feb 10;8:588176. doi: 10.3389/fvets.2021.588176 (PMC7928357; doi:10.3389/fvets.2021.588176)
Supplement: Supplementary file 1 [file Data_Sheet_1.zip › S28_file.docx]

**File S16: OpenBUGS Script for the latent class analysis in the Bayesian framework**

######################################################

#Conditionally dependent Bayesian latent class analysis

#author: Clara Schoneberg

#date: 26th February 2020

#######################################################

model

{

#Multinomial distributions

r1[1:8] ~ dmulti(p1[1:8], n1)

#### Probabilities of tests to be positive in the population

# p(1 1 1): Probability of all three tests to be positive

p1[1]<-(pr)*(se[1]*se[2]*se[3] + se[1]*a23 + se[2]*a13+ se[3]*a12 + a123) + (1-pr)*((1-sp[1])*(1-sp[2])*(1-sp[3]) + (1-sp[1])*b23 + (1-sp[2])*b13 + (1-sp[3])*b12 - b123)

# p(1 1 0): Probability of first two test to be positive and the third test to be negative

p1[2] <- pr*(se[1]*se[2]*(1-se[3]) - se[1]*a23 - se[2]*a13 + (1-se[3])*a12 - a123) + (1-pr)*((1-sp[1])*(1-sp[2])*sp[3] - (1-sp[1])*b23 - (1-sp[2])*b13 + sp[3]*b12 + b123)

# p(1 0 1): Probability of first and third test to be positive but the second test negative

p1[3] <- pr*(se[1]*(1-se[2])*se[3] - se[1]*a23 + (1-se[2])*a13 - se[3]*a12-a123) + (1-pr)*((1-sp[1])*sp[2]*(1-sp[3]) - (1-sp[1])*b23+sp[2]*b13 - (1-sp[3])*b12 + b123)

# p(1 0 0):Probability of first test to be positive but the second and third tests negative

p1[4] <- pr*(se[1]*(1-se[2])*(1-se[3]) + se[1]*a23 - (1-se[2])*a13 - (1-se[3])*a12+a123) + (1-pr)*((1-sp[1])*sp[2]*sp[3] + (1-sp[1])*b23 - sp[2]*b13 - sp[3]*b12 - b123)

# p(0 1 1):Probability of first test to be negative but the second and third tests positive

p1[5] <- pr*((1-se[1])*se[2]*se[3] + (1-se[1])*a23 - se[2]*a13 - se[3]*a12-a123) + (1-pr)*(sp[1]*(1-sp[2])*(1-sp[3]) + sp[1]*b23 - (1-sp[2])*b13 - (1-sp[3])*b12 + b123)

# p(0 1 0):Probability of first and third test to be negative but the second test positive

p1[6] <- pr*((1-se[1])*se[2]*(1-se[3]) - (1-se[1])*a23 + se[2]*a13 - (1-se[3])*a12+a123) + (1-pr)*(sp[1]*(1-sp[2])*sp[3] - sp[1]*b23 + (1-sp[2])*b13 - sp[3]*b12 - b123)

#p(0 0 1): Probability of first two test to be negative and the third test to be positive

p1[7] <- pr*((1-se[1])*(1-se[2])*se[3] - (1-se[1])*a23 - (1-se[2])*a13 + se[3]*a12 + a123) + (1-pr)*(sp[1]*sp[2]*(1-sp[3]) - sp[1]*b23 - sp[2]*b13 + (1-sp[3])*b12 - b123)

#p(0 0 0): Probability of all three tests to be negative

p1[8] <- pr*((1-se[1])*(1-se[2])*(1-se[3]) + (1-se[1])*a23 + (1-se[2])*a13 + (1-se[3])*a12-a123) + (1-pr)*(sp[1]*sp[2]*sp[3] + sp[1]*b23 + sp[2]*b13 + sp[3]*b12 + b123)

#### Prior information for se and sp of three tests

####Scenario 1

#Starting Information 1

#pr ~ dbeta(999.99, 2333.33)

#se[1] ~ dbeta(999.99, 111.11)

#se[2] ~ dbeta(999.99, 176.47)

#se[3] ~ dbeta(999.99, 111.11)

#sp[1] ~ dbeta(999.99, 52.63)

#sp[2] ~ dbeta(999.99, 52.63)

#sp[3] ~ dbeta(999.99, 10.10)

#Starting Information 2

#pr ~ dbeta(96.55334, 225.2911)

#se[1] ~dbeta(128.0977, 14.23308)

#se[2] ~ dbeta(167.8375, 29.61838)

#se[3] ~ dbeta(128.0977, 14.23308)

#sp[1] ~ dbeta(115.8176, 6.095662)

#sp[2] ~ dbeta(115.8176, 6.095662)

#sp[3] ~ dbeta(81.84448, 0.8267119)

#Starting Information 3

#pr ~ dbeta(96.55334, 225.2911)

#se[1] ~dbeta(128.0977, 14.23308)

#se[2] ~ dbeta(167.8375, 29.61838)

#se[3] ~ dbeta(128.0977, 14.23308)

#sp[1] ~ dbeta(115.8176, 6.095662)

#sp[2] ~ dbeta(115.8176, 6.095662)

#sp[3] ~ dbeta(81.84448, 0.8267119)

#Starting Information 4

#pr ~ dbeta(999.99, 1857.14)

#se[1] ~ dbeta(999.99, 111.11)

#se[2] ~ dbeta(999.99, 111.11)

#se[3] ~ dbeta(999.99, 111.11)

#sp[1] ~ dbeta(999.99, 52.63)

#sp[2] ~ dbeta(999.99, 52.63)

#sp[3] ~ dbeta(999.99, 10.10)

#Starting Information 5

#pr ~ dbeta(999.99, 1500)

#se[1] ~ dbeta(999.99, 86.96)

#se[2] ~ dbeta(999.99, 204.82)

#se[3] ~ dbeta(999.99, 63.83)

#sp[1] ~ dbeta(999.99, 86.96)

#sp[2] ~ dbeta(999.99, 75.27)

#sp[3] ~ dbeta(999.99, 20.41)

#Starting Information 6

#pr ~ dbeta(999.99, 1500)

#se[1] ~ dbeta(999.99, 86.96)

#se[2] ~ dbeta(999.99, 204.82)

#se[3] ~ dbeta(999.99, 63.83)

#sp[1] ~ dbeta(999.99, 86.96)

#sp[2] ~ dbeta(999.99, 75.27)

#sp[3] ~ dbeta(999.99, 20.41)

#Starting Information 7

#pr ~ dbeta(1,1)

#se[1] ~ dbeta(1,1)

#se[2] ~ dbeta(1,1)

#se[3] ~ dbeta(1,1)

#sp[1] ~ dbeta(1,1)

#sp[2] ~ dbeta(1,1)

#sp[3] ~ dbeta(1,1)

#Starting Information 8

#pr ~ dbeta(199.9748, 599.9243)

#se[1] ~ dbeta(463.86, 81.86)

#se[2] ~ dbeta(463.86, 81.86)

#se[3] ~ dbeta(463.86, 81.86)

#sp[1] ~ dbeta(349.33, 38.81)

#sp[2] ~ dbeta(88.45, 4.66)

#sp[3] ~ dbeta(265.1052, 19.95415)

#Starting Information 9

pr ~ dbeta(135.33, 348.00)

se[1] ~ dbeta(225.29, 96.55)

se[2] ~ dbeta(225.29, 96.55)

se[3] ~ dbeta(9.392, 0.095)

sp[1] ~ dbeta(196.93, 49.23)

sp[2] ~ dbeta(196.93, 49.23)

sp[3] ~ dbeta(9.392, 0.095)

####Scenario 2

#Starting Information 1
#pr ~ dbeta(999.9999, 1500)
#se[1] ~ dbeta(999.9999, 111.1111)
#se[2] ~ dbeta(999.9999, 428.5714)
#se[3] ~ dbeta(999.9999, 538.4615)
#sp[1] ~ dbeta(999.9999, 10.10101)
#sp[2] ~ dbeta(999.9999, 250)
#sp[3] ~ dbeta(999.9999, 176.4706)

#Starting Information 2
#pr ~ dbeta(146.9934, 220.4901)
#se[1] ~dbeta(128.0977, 14.23308)
#se[2] ~ dbeta(225.2911, 96.55334)
#se[3] ~ dbeta(226.4941, 121.9583)
#sp[1] ~ dbeta(23.16483, 0.2339882)
#sp[2] ~ dbeta(196.9316, 49.2329)
#sp[3] ~ dbeta(167.8375, 29.61838)

#Starting Information 3
pr ~ dbeta(146.9934, 220.4901)
se[1] ~dbeta(128.0977, 14.23308)
se[2] ~ dbeta(225.2911, 96.55334)
se[3] ~ dbeta(226.4941, 121.9583)
sp[1] ~ dbeta(23.16483, 0.2339882)
sp[2] ~ dbeta(196.9316, 49.2329)
sp[3] ~ dbeta(167.8375, 29.61838)

#Starting Information 4
#pr ~ dbeta(96.55334, 225.2911)
#se[1] ~ dbeta(167.8375, 29.61838)
#se[2] ~ dbeta(215.7842, 71.92806)
#se[3] ~ dbeta(222.5449, 86.54525)
#sp[1] ~ dbeta(32.73211, 0.3306274)
#sp[2] ~ dbeta(167.8375, 29.61838)
#sp[3] ~ dbeta(136.8737, 16.91698)

#Starting Information 5
#pr ~ dbeta(690.7717, 844.2765)
#se[1] ~ dbeta(99.68398, 7.503095)
#se[2] ~ dbeta(226.8656, 116.8702)
#se[3] ~ dbeta(225.9638, 138.494)
#sp[1] ~ dbeta(32.73211, 0.3306274)
#sp[2] ~ dbeta(215.7842, 71.92806)
#sp[3] ~ dbeta(136.8737, 16.91698)

#Starting Information 6
#pr ~ dbeta(690.7717, 844.2765)
#se[1] ~ dbeta(99.68398, 7.503095)
#se[2] ~ dbeta(226.8656, 116.8702)
#se[3] ~ dbeta(225.9638, 138.494)
#sp[1] ~ dbeta(32.73211, 0.3306274)
#sp[2] ~ dbeta(215.7842, 71.92806)
#sp[3] ~ dbeta(136.8737, 16.91698)

#Starting Information 7 (const. in all scenarios)
#pr ~ dbeta(1,1)
#se[1] ~ dbeta(1,1)
#se[2] ~ dbeta(1,1)
#se[3] ~ dbeta(1,1)
#sp[1] ~ dbeta(1,1)
#sp[2] ~ dbeta(1,1)
#sp[3] ~ dbeta(1,1)

#Starting Information 8
#pr ~ dbeta(96.55334, 225.2911)
#se[1] ~ dbeta(128.0977, 14.23308)
#se[2] ~ dbeta(196.9316, 49.2329)
#se[3] ~ dbeta(196.9316, 49.2329)
#sp[1] ~ dbeta(32.73211, 0.3306274)
#sp[2] ~ dbeta(128.0977, 14.23308)
#sp[3] ~ dbeta(128.0977, 14.23308)

#Starting Information 9
#pr ~ dbeta(9144.5393, 221.383)
#se[1] ~ dbeta(225.6786, 98.57228)
#se[2] ~ dbeta(132.5347, 15.54876)
#se[3] ~ dbeta(171.2266, 31.40843)
#sp[1] ~ dbeta(167.8375, 29.61838)
#sp[2] ~ dbeta(106.548, 8.88862)
#sp[3] ~ dbeta(9.366606, 0.2599161)

####Scenario 3

#Starting Information 1
#pr ~ dbeta(40.92541, 1323.255)
#se[1] ~ dbeta(999.9999, 111.1111)
#se[2] ~ dbeta(999.9999, 428.5714)
#se[3] ~ dbeta(999.9999, 538.4615)
#sp[1] ~ dbeta(999.9999, 10.10101)
#sp[2] ~ dbeta(999.9999, 250)
#sp[3] ~ dbeta(999.9999, 176.4706)

#Starting Information 2
#pr ~ dbeta(2.092657, 67.66259)
#se[1] ~dbeta(128.0977, 14.23308)
#se[2] ~ dbeta(225.2911, 96.55334)
#se[3] ~ dbeta(226.4941, 121.9583)
#sp[1] ~ dbeta(23.16483, 0.2339882)
#sp[2] ~ dbeta(196.9316, 49.2329)
#sp[3] ~ dbeta(167.8375, 29.61838)

#Starting Information 3
#pr ~ dbeta(2.092657, 67.66259)
#se[1] ~dbeta(128.0977, 14.23308)
#se[2] ~ dbeta(225.2911, 96.55334)
#se[3] ~ dbeta(226.4941, 121.9583)
#sp[1] ~ dbeta(23.16483, 0.2339882)
#sp[2] ~ dbeta(196.9316, 49.2329)
#sp[3] ~ dbeta(167.8375, 29.61838)

#Starting Information 4
#pr ~ dbeta(4.459058, 441.4468)
#se[1] ~ dbeta(167.8375, 29.61838)
#se[2] ~ dbeta(215.7842, 71.92806)
#se[3] ~ dbeta(222.5449, 86.54525)
#sp[1] ~ dbeta(32.73211, 0.3306274)
#sp[2] ~ dbeta(167.8375, 29.61838)
#sp[3] ~ dbeta(136.8737, 16.91698)

#Starting Information 5
pr ~ dbeta(2.408616, 57.80679)
se[1] ~ dbeta(999.9999, 75.26881)
se[2] ~ dbeta(999.9999, 515.1515)
se[3] ~ dbeta(999.9999, 612.9032)
sp[1] ~ dbeta(32.73211, 0.3306274)
sp[2] ~ dbeta(999.9999, 333.3333 )
sp[3] ~ dbeta(205.6566, 58.00571)

#Starting Information 6
#pr ~ dbeta(15.54128, 502.5013)
#se[1] ~ dbeta(196.9316, 49.2329)
#se[2] ~ dbeta(196.9316, 49.2329)
#se[3] ~ dbeta(196.9316, 49.2329)
#sp[1] ~ dbeta(196.9316, 49.2329)
#sp[2] ~ dbeta(196.9316, 49.2329)
#sp[3] ~ dbeta(196.9316, 49.2329)

#Starting Information 7 (const. in all scenarios)
#pr ~ dbeta(1,1)
#se[1] ~ dbeta(1,1)
#se[2] ~ dbeta(1,1)
#se[3] ~ dbeta(1,1)
#sp[1] ~ dbeta(1,1)
#sp[2] ~ dbeta(1,1)
#sp[3] ~ dbeta(1,1)

#Starting Information 8
#pr ~ dbeta(175.4921, 4211.81)
#se[1] ~ dbeta(36.66453, 4.073837)
#se[2] ~ dbeta(49.47791, 12.36948)
#se[3] ~ dbeta(49.47791, 12.36948)
#sp[1] ~ dbeta(32.73211, 0.3306274)
#sp[2] ~ dbeta(34.98895, 3.887661)
#sp[3] ~ dbeta(34.98895, 3.887661)

#Starting Information 9
#pr ~ dbeta(15.72836, 770.6895)
#se[1] ~ dbeta(220.4901, 146.9934)
#se[2] ~ dbeta(131.655, 15.28139)
#se[3] ~ dbeta(87.58409, 16.31163)
#sp[1] ~ dbeta(105.4639, 1.933148)
#sp[2] ~ dbeta(105.5744, 8.683609)
#sp[3] ~ dbeta(33.79391, 0.9377551)

#Scenario4

#Starting Information 1
#pr ~ dbeta(999.9999, 1500)
#se[1] ~ dbeta(999.9999, 250)
#se[2] ~ dbeta(999.9999, 515.1515)
#se[3] ~ dbeta(999.9999, 428.5714)
#sp[1] ~ dbeta(999.9999, 52.63157)
#sp[2] ~ dbeta(999.9999, 176.4706)
#sp[3] ~ dbeta(999.9999, 136.3636)


#Starting Information 2
#pr ~ dbeta(36.3567, 54.53505)
#se[1] ~dbeta(49.47791, 12.36948)
#se[2] ~ dbeta(56.18713, 28.94489)
#se[3] ~ dbeta(55.89292, 23.95411)
#sp[1] ~ dbeta(28.36228, 1.492751)
#sp[2] ~ dbeta(43.04593, 7.596341)
#sp[3] ~ dbeta(38.26855, 5.218439)

#Starting Information 3
#pr ~ dbeta(36.3567, 54.53505)
#se[1] ~dbeta(49.47791, 12.36948)
#se[2] ~ dbeta(56.18713, 28.94489)
#se[3] ~ dbeta(55.89292, 23.95411)
#sp[1] ~ dbeta(28.36228, 1.492751)
#sp[2] ~ dbeta(43.04593, 7.596341)
#sp[3] ~ dbeta(38.26855, 5.218439)


#Starting Information 4
pr ~ dbeta(26.44229, 56.18986)
se[1] ~ dbeta(54.53504, 36.3567)
se[2] ~ dbeta(54.53504, 36.3567)
se[3] ~ dbeta(54.53504, 36.3567)
sp[1] ~ dbeta(49.47791, 12.36948)
sp[2] ~ dbeta(49.47791, 12.36948)
sp[3] ~ dbeta(49.47791, 12.36948)

#Starting Information 5
#pr ~ dbeta(170.5098, 208.4009)
#se[1] ~ dbeta(599.9243, 199.9748)
#se[2] ~ dbeta(999.9999, 587.3015)
#se[3] ~ dbeta(999.9999, 515.1515)
#sp[1] ~ dbeta(136.8737, 16.91698)
#sp[2] ~ dbeta(49.47791, 12.36948)
#sp[3] ~ dbeta(48.36091, 11.34392)

#Starting Information 6
#pr ~ dbeta(36.3567, 54.53505)
#se[1] ~ dbeta(49.47791, 12.36948)
#se[2] ~ dbeta(49.47791, 12.36948)
#se[3] ~ dbeta(49.47791, 12.36948)
#sp[1] ~ dbeta(49.47791, 12.36948)
#sp[2] ~ dbeta(49.47791, 12.36948)
#sp[3] ~ dbeta(49.47791, 12.36948)

#Starting Information 7 (const. in all scenarios)
#pr ~ dbeta(1,1)
#se[1] ~ dbeta(1,1)
#se[2] ~ dbeta(1,1)
#se[3] ~ dbeta(1,1)
#sp[1] ~ dbeta(1,1)
#sp[2] ~ dbeta(1,1)
#sp[3] ~ dbeta(1,1)

#Starting Information 8
#pr ~ dbeta(23.95411, 55.89292)
#se[1] ~ dbeta(220.4901, 146.9934)
#se[2] ~ dbeta(220.4901, 146.9934)
#se[3] ~ dbeta(196.9316, 49.2329)
#sp[1] ~ dbeta(128.0977, 14.23308)
#sp[2] ~ dbeta(128.0977, 14.23308)
#sp[3] ~ dbeta(128.0977, 14.23308)

#Starting Information 9
#pr ~ dbeta(539.7481, 899.5802)
#se[1] ~ dbeta(201.4962, 53.56229)
#se[2] ~ dbeta(199.2649, 51.38277)
#se[3] ~ dbeta(162.9118, 27.18365)
#sp[1] ~ dbeta(115.1857, 10.83805)
#sp[2] ~ dbeta(118.9445, 11.76375)
#sp[3] ~ dbeta(80.2545, 4.13509)

#Scenario5

#Starting Information 1
#pr ~ dbeta(999.9999, 4000)
#se[1] ~ dbeta(999.9999, 388.8889)
#se[2] ~ dbeta(999.9999, 538.4615)
#se[3] ~ dbeta(999.9999, 30.92783)
#sp[1] ~ dbeta(770.6885, 15.72834)
#sp[2] ~ dbeta(463.1652, 4.678436)
#sp[3] ~ dbeta(770.6885, 15.72834)


#Starting Information 2
#pr ~ dbeta(12.36947, 49.4779)
#se[1] ~dbeta(55.2822, 21.49863)
#se[2] ~ dbeta(56.07807, 30.19588)
#se[3] ~ dbeta(20.9666, 0.6484516)
#sp[1] ~ dbeta(15.80247, 0.3224994)
#sp[2] ~ dbeta(4.651567, 0.04698552)
#sp[3] ~ dbeta(15.80247, 0.3224994)


#Starting Information 3
#pr ~ dbeta(12.36947, 49.4779)
#se[1] ~dbeta(55.2822, 21.49863)
#se[2] ~ dbeta(56.07807, 30.19588)
#se[3] ~ dbeta(20.9666, 0.6484516)
#sp[1] ~ dbeta(15.80247, 0.3224994)
#sp[2] ~ dbeta(4.651567, 0.04698552)
#sp[3] ~ dbeta(15.80247, 0.3224994)


#Starting Information 4
#pr ~ dbeta(734.9035, 4164.453)
#se[1] ~ dbeta(999.9999, 298.7013)
#se[2] ~ dbeta(626.8496, 268.6498)
#se[3] ~ dbeta(23.63559 0.482359)
#sp[1] ~ dbeta(9.392057, 0.0948692)
#sp[2] ~ dbeta(40.04885, 0.04008893)
#sp[3] ~ dbeta(9.392057 0.09486926)


#Starting Information 5
#pr ~ dbeta(12.36947, 49.4779)
#se[1] ~ dbeta(24.52117, 13.20371)
#se[2] ~ dbeta(24.52117, 13.20371)
#se[3] ~ dbeta(80.5077, 4.237247)
#sp[1] ~ dbeta(80.5077, 4.237247)
#sp[2] ~ dbeta(80.5077, 4.237247)
#sp[3] ~ dbeta(80.5077, 4.237247)

#Starting Information 6
#pr ~ dbeta(58.8104, 196.887)
#se[1] ~ dbeta(55.89292, 23.95411)
#se[2] ~ dbeta(24.59581, 11.05029)
#se[3] ~ dbeta(80.5077, 4.237247)
#sp[1] ~ dbeta(9.392057, 0.09486926)
#sp[2] ~ dbeta(9.392057, 0.09486926)
#sp[3] ~ dbeta(9.392057, 0.09486926)

#Starting Information 7 (const. in all scenarios)
#pr ~ dbeta(1,1)
#se[1] ~ dbeta(1,1)
#se[2] ~ dbeta(1,1)
#se[3] ~ dbeta(1,1)
#sp[1] ~ dbeta(1,1)
#sp[2] ~ dbeta(1,1)
#sp[3] ~ dbeta(1,1)

#Starting Information 8
#pr ~ dbeta(96.55334, 225.2911)
#se[1] ~ dbeta(167.8375, 29.61838)
#se[2] ~ dbeta(196.9316, 49.2329)
#se[3] ~ dbeta(225.2911, 96.55334)
#sp[1] ~ dbeta(9.392057, 0.09486926)
#sp[2] ~ dbeta(9.392057, 0.09486926)
#sp[3] ~ dbeta(34.98895, 3.887661)

#Starting Information 9
pr ~ dbeta(816.9388, 4321.041)
se[1] ~ dbeta(113.287, 10.38876)
se[2] ~ dbeta(180.7491, 37.0209)
se[3] ~ dbeta(46.05686, 0.7488921)
sp[1] ~ dbeta(36.37767, 0.6668005)
sp[2] ~ dbeta(18.32756, 0.1478029)
sp[3] ~ dbeta(93.72634, 6.408638)

#### Lower and upper limits

#sensitivities

ll1 <- max(-(1-se[1])*(1-se[2]), -se[1]*se[2])

ul1 <- min(se[1]*(1-se[2]),(1-se[1])*se[2])

a12 ~ dunif(ll1,ul1)

ll2 <- max(-(1-se[1])*(1-se[3]), -se[1]*se[3])

ul2 <- min(se[1]*(1-se[3]),(1-se[1])*se[3])

a13 ~ dunif(ll2,ul2)

ll3 <- max(-(1-se[2])*(1-se[3]), -se[2]*se[3])

ul3 <- min(se[2]*(1-se[3]),(1-se[2])*se[3])

a23 ~ dunif(ll3,ul3)

ll71 <- -(se[1]*se[2]*se[3]+se[1]*a23+se[2]*a13+se[3]*a12)

ll72 <- -((1-se[1])*(1-se[2])*se[3]-(1-se[1])*a23-(1-se[2])*a13+se[3]*a12)

ll73 <- -((1-se[1])*se[2]*(1-se[3])-(1-se[1])*a23+se[2]*a13-(1-se[3])*a12)

ll74 <- -(se[1]*(1-se[2])*(1-se[3])+se[1]*a23-(1-se[2])*a13-(1-se[3])*a12)

ll7 <- max(max(ll71, ll72), max(ll73, ll74))

ul71 <- (1-se[1])*se[2]*se[3]+(1-se[1])*a23-se[2]*a13-se[3]*a12

ul72 <- se[1]*(1-se[2])*se[3]-se[1]*a23+(1-se[2])*a13-se[3]*a12

ul73 <- se[1]*se[2]*(1-se[3])-se[1]*a23-se[2]*a13+(1-se[3])*a12

ul74 <- (1-se[1])*(1-se[2])*(1-se[3])+(1-se[1])*a23+(1-se[2])*a13+(1-se[3])*a12

ul7 <- min(min(ul71, ul72), min(ul73, ul74))

l1l <- min(ll7,ul7)

u1l <- max(ll7,ul7)

a123 ~ dunif(l1l,u1l)

#### specificities

ll4 <- max(-(1-sp[1])*(1-sp[2]), -sp[1]*sp[2])

ul4 <- min(sp[1]*(1-sp[2]),(1-sp[1])*sp[2])

b12 ~ dunif(ll4,ul4)

ll5 <- max(-(1-sp[1])*(1-sp[3]), -sp[1]*sp[3])

ul5 <- min(sp[1]*(1-sp[3]),(1-sp[1])*sp[3])

b13 ~ dunif(ll5,ul5)

ll6 <- max(-(1-sp[2])*(1-sp[3]), -sp[2]*sp[3])

ul6 <- min(sp[2]*(1-sp[3]),(1-sp[2])*sp[3])

b23 ~ dunif(ll6,ul6)

ll81 <- -(sp[1]*sp[2]*sp[3]+sp[1]*b23+sp[2]*b13+sp[3]*b12)

ll82 <- -((1-sp[1])*(1-sp[2])*sp[3]-(1-sp[1])*b23-(1-sp[2])*b13+sp[3]*b12)

ll83 <- -((1-sp[1])*sp[2]*(1-sp[3])-(1-sp[1])*b23+sp[2]*b13-(1-sp[3])*b12)

ll84 <- -(sp[1]*(1-sp[2])*(1-sp[3])+sp[1]*b23-(1-sp[2])*b13-(1-sp[3])*b12)

ll8<- max(max(ll81, ll82), max(ll83, ll84))

ul81 <- (1-sp[1])*sp[2]*sp[3]+(1-sp[1])*b23-sp[2]*b13-sp[3]*b12

ul82 <- sp[1]*(1-sp[2])*sp[3]-sp[1]*b23+(1-sp[2])*b13-sp[3]*b12

ul83 <- sp[1]*sp[2]*(1-sp[3])-sp[1]*b23-sp[2]*b13+(1-sp[3])*b12

ul84 <- (1-sp[1])*(1-sp[2])*(1-sp[3])+(1-sp[1])*b23+(1-sp[2])*b13+(1-sp[3])*b12

ul8<- min(min(ul81, ul82), min(ul83, ul84))

l2l<- min(ll8,ul8)

u2l <- max(ll8,ul8)

b123 ~ dunif(l2l,u2l)

# Standardized dependencies

posAbh12 <- a12/(sqrt(se[1]*(1-se[1]))*sqrt(se[2]*(1-se[2])))

posAbh23 <- a23/(sqrt(se[2]*(1-se[2]))*sqrt(se[3]*(1-se[3])))

posAbh13 <- a13/(sqrt(se[1]*(1-se[1]))*sqrt(se[3]*(1-se[3])))

negAbh12 <- b12/(sqrt(sp[1]*(1-sp[1]))*(sp[2]*(1-sp[2])))

negAbh23 <- b23/(sqrt(sp[2]*(1-sp[2]))*(sp[3]*(1-sp[3])))

negAbh13 <- b13/(sqrt(sp[1]*(1-sp[1]))*(sp[3]*(1-sp[3])))

}

#Data

#Simulation1:

list(r1=c(2066,247,368,369,232,355,104,6259),n1=1000)

#1. Starting value:

list(pr=0.30, se=c(0.90,0.85,0.90),sp=c(0.95,0.95,0.99),a12=0.0 ,a13=0.0 ,a23=0.0,a123= 0.0,b12=0.0 ,b13=0.0 ,b23= 0.0,b123= 0.0,r11=c(205,27,36,37,23,29,17,626))

#2. Starting value:

list(pr=0.30, se=c(0.90,0.85,0.90),sp=c(0.95,0.95,0.99),a12=0.0 ,a13=0.0 ,a23=0.030,a123= 0.001,b12=0.0 ,b13=0.0 ,b23= 0.009,b123= 0.0001,r11=c(205,27,36,37,23,29,17,626))

#3. Starting value:

list(pr=0.30, se=c(0.90,0.85,0.90),sp=c(0.95,0.95,0.99),a12=0.01 ,a13=0.01 ,a23=0.01,a123= 0.0,b12=0.003 ,b13=0.003 ,b23= 0.003,b123= -0.003,r11=c(205,27,36,37,23,29,17,626))

#4. Starting value:

list(pr=0.35, se=c(0.90,0.90,0.90),sp=c(0.95,0.95,0.95),a12=0.0 ,a13=0.0 ,a23=0.0,a123= 0.0,b12=0.0 ,b13=0.0 ,b23= 0.0,b123= 0.0,r11=c(205,27,36,37,23,29,17,626))

#5. Starting value:

list(pr=0.40, se=c(0.92,0.83,0.94),sp=c(0.92,0.93,0.98),a12=0.0 ,a13=0.0 ,a23=0.0,a123= 0.0,b12=0.0 ,b13=0.0 ,b23= 0.0,b123= 0.0,r11=c(205,27,36,37,23,29,17,626))

#6. Starting value:

list(pr=0.40, se=c(0.92,0.83,0.94),sp=c(0.92,0.93,0.98),a12=0.0 ,a13=0.0 ,a23=0.03,a123= 0.001,b12=0.0 ,b13=0.0 ,b23= 0.009,b123= 0.0001,r11=c(205,27,36,37,23,29,17,626))

#7. Starting value:

list(pr=0.50, se=c(0.50,0.50,0.50),sp=c(0.50,0.50,0.50),a12=0.0 ,a13=0.0 ,a23=0.0,a123= 0.0,b12=0.0 ,b13=0.0 ,b23= 0.0,b123= 0.0,r11=c(205,27,36,37,23,29,17,626))

#8. Starting value:

list(pr=0.25, se=c(0.85,0.85,0.85),sp=c(0.90,0.95,0.93),a12=0.0 ,a13=0.01 ,a23=0.0,a123= 0.0,b12=0.0 ,b13=0.001 ,b23= 0.0,b123= 0.0,r11=c(205,27,36,37,23,29,17,626))

#9. Starting value:

list(pr=0.28, se=c(0.70,0.70,0.99),sp=c(0.80,0.80,0.99),a12=0.04 ,a13=0.0 ,a23=0.0,a123= -0.001,b12=0.009 ,b13=0.0 ,b23= 0.0,b123= -0.001,r11=c(205,27,36,37,23,29,17,626))

#Simulation2:
list(r1=c(2082,450,267,861,918,550,233,4639),n1=10000)

#1. Starting value:
list(pr=0.40, se=c(0.90,0.70,0.65),sp=c(0.99,0.80,0.85),a12=0.0 ,a13=0.0 ,a23=0.121,a123= 0.0,b12=0.0 ,b13=0.0 ,b23= 0.086,b123= 0.0,r11=c(2082,450,267,861,918,550,233,4639))

#2. Starting value:
list(pr=0.40, se=c(0.90,0.70,0.65),sp=c(0.99,0.80,0.85),a12=0.0 ,a13=0.0 ,a23=0.140,a123= 0.000,b12=0.0 ,b13=0.0 ,b23= 0.095,b123= 0.0,r11=c(2082,450,267,861,918,550,233,4639))

#3. Starting value:
list(pr=0.40, se=c(0.90,0.70,0.65),sp=c(0.99,0.80,0.85),a12=0.00 ,a13=0.00 ,a23=0.09,a123= 0.0,b12=0.0 ,b13=0.0 ,b23= 0.065,b123=0.0,r11=c(2082,450,267,861,918,550,233,4639))

#4. Starting value:
list(pr=0.30, se=c(0.85,0.75,0.72),sp=c(0.99,0.85,0.89),a12=0.0 ,a13=0.0 ,a23=0.117,a123= 0.0,b12=0.0 ,b13=0.0 ,b23= 0.067,b123= 0.0,r11=c(2082,450,267,861,918,550,233,4639))

#5. Starting value:
list(pr=0.45, se=c(0.93,0.66,0.62),sp=c(0.99,0.75,0.89),a12=0.0 ,a13=0.0 ,a23=0.138,a123= 0.0,b12=0.0 ,b13=0.0 ,b23= 0.082,b123= 0.0,r11=c(2082,450,267,861,918,550,233,4639))

#6. Starting value:
list(pr=0.45, se=c(0.93,0.66,0.62),sp=c(0.99,0.75,0.89),a12=0.0 ,a13=0.0 ,a23=0.11,a123= 0.00,b12=0.0 ,b13=0.0 ,b23= 0.080,b123= 0.0001,r11=c(2082,450,267,861,918,550,233,4639))

#7. Starting value:
list(pr=0.50, se=c(0.50,0.50,0.50),sp=c(0.50,0.50,0.50),a12=0.0 ,a13=0.0 ,a23=0.0,a123= 0.0,b12=0.0 ,b13=0.0 ,b23= 0.0,b123= 0.0,r11=c(2082,450,267,861,918,550,233,4639))

#8. Starting value:
list(pr=0.30, se=c(0.90,0.80,0.80),sp=c(0.99,0.90,0.90),a12=0.0 ,a13=0.0 ,a23=0.01,a123= 0.0,b12=0.0 ,b13=0.001 ,b23= 0.001,b123= 0.0,r11=c(2082,450,267,861,918,550,233,4639))

#9. Starting value:
list(pr=0.395, se=c(0.696,0.895,0.845),sp=c(0.85,0.923,0.973),a12=0.04 ,a13=0.0 ,a23=0.0,a123= -0.001,b12=0.009 ,b13=0.0 ,b23= 0.0,b123= -0.001,r11=c(2082,450,267,861,918,550,233,4639))

#Simulation3:
list(r1=c(167,42,23,135,1128,813,332,7360),n1=10000)

#1. Starting value:
list(pr=0.03, se=c(0.90,0.70,0.65),sp=c(0.99,0.80,0.85),a12=0.0 ,a13=0.0 ,a23=0.121,a123= 0.0,b12=0.0 ,b13=0.0 ,b23= 0.086,b123= 0.0,r11=c(167,42,23,135,1128,813,332,7360))

#2. Starting value:
list(pr=0.03, se=c(0.90,0.70,0.65),sp=c(0.99,0.80,0.85),a12=0.0 ,a13=0.0 ,a23=0.140,a123= 0.000,b12=0.0 ,b13=0.0 ,b23= 0.095,b123= 0.0,r11=c(167,42,23,135,1128,813,332,7360))

#3. Starting value:
list(pr=0.03, se=c(0.90,0.70,0.65),sp=c(0.99,0.80,0.85),a12=0.00 ,a13=0.00 ,a23=0.09,a123= 0.0,b12=0.0 ,b13=0.0 ,b23= 0.065,b123=0.0,r11=c(167,42,23,135,1128,813,332,7360))

#4. Starting value:
list(pr=0.01, se=c(0.85,0.75,0.72),sp=c(0.99,0.85,0.89),a12=0.0 ,a13=0.0 ,a23=0.117,a123= 0.0,b12=0.0 ,b13=0.0 ,b23= 0.067,b123= 0.0,r11=c(167,42,23,135,1128,813,332,7360))

#5. Starting value:
list(pr=0.04, se=c(0.93,0.66,0.62),sp=c(0.99,0.75,0.78),a12=0.0 ,a13=0.0 ,a23=0.138,a123= 0.0,b12=0.0 ,b13=0.0 ,b23= 0.108,b123= 0.0,r11=c(167,42,23,135,1128,813,332,7360))
#6. Starting value:
list(pr=0.03, se=c(0.80,0.80,0.80),sp=c(0.80,0.80,0.80),a12=0.0 ,a13=0.0 ,a23=0.11,a123= 0.00,b12=0.0 ,b13=0.0 ,b23= 0.095,b123= 0.0001,r11=c(167,42,23,135,1128,813,332,7360))

#7. Starting value:
list(pr=0.50, se=c(0.50,0.50,0.50),sp=c(0.50,0.50,0.50),a12=0.0 ,a13=0.0 ,a23=0.0,a123= 0.0,b12=0.0 ,b13=0.0 ,b23= 0.0,b123= 0.0,r11=c(167,42,23,135,1128,813,332,7360))

#8. Starting value:
list(pr=0.04, se=c(0.90,0.80,0.80),sp=c(0.99,0.90,0.90),a12=0.0 ,a13=0.0 ,a23=0.01,a123= 0.0,b12=0.0 ,b13=0.001 ,b23= 0.001,b123= 0.0,r11=c(167,42,23,135,1128,813,332,7360))

#9. Starting value:
list(pr=0.02, se=c(0.60,0.896,0.843),sp=c(0.982,0.924,0.973),a12=0.01 ,a13=0.01 ,a23=0.08,a123= -0.007,b12=0.008 ,b13=0.001 ,b23= 0.0,b123= 0.0,r11=c(167,42,23,135,1128,813,332,7360))

#Simulation4:
list(r1=c(2020,382,545,553,561,577,393,4969),n1=10000)

#1. Starting value:
list(pr=0.40, se=c(0.80,0.66,0.70),sp=c(0.95,0.85,0.88),a12=0.038 ,a13=0.046 ,a23=0.087,a123= -0.004,b12=0.0156 ,b13=0.018 ,b23= 0.046,b123= -0.0013,r11=c(2020,382,545,553,561,577,393,4969))

#2. Starting value:
list(pr=0.40, se=c(0.80,0.66,0.70),sp=c(0.95,0.85,0.88),a12=0.048 ,a13=0.055 ,a23=0.097,a123= -0.005,b12=0.02 ,b13=0.022 ,b23= 0.05,b123= -0.002,r11=c(2020,382,545,553,561,577,393,4969))

#3. Starting value:
list(pr=0.40, se=c(0.80,0.66,0.70),sp=c(0.95,0.85,0.88),a12=0.025 ,a13=0.033 ,a23=0.062,a123= -0.003,b12=0.01 ,b13=0.012 ,b23= 0.032,b123=-0.001,r11=c(2020,382,545,553,561,577,393,4969))

#4. Starting value:
list(pr=0.35, se=c(0.85,0.73,0.75),sp=c(0.98,0.88,0.92),a12=0.0317 ,a13=0.0387 ,a23=0.0769,a123= -0.003,b12=0.009 ,b13=0.01 ,b23= 0.035,b123= -0.001,r11=c(2020,382,545,553,561,577,393,4969))

#5. Starting value:
list(pr=0.45, se=c(0.60,0.60,0.60),sp=c(0.80,0.80,0.80),a12=0.048 ,a13=0.060 ,a23=0.096,a123= -0.006,b12=0.032 ,b13=0.040 ,b23= 0.064,b123= -0.003,r11=c(2020,382,545,553,561,577,393,4969))

#6. Starting value:
list(pr=0.40, se=c(0.80,0.80,0.80),sp=c(0.80,0.80,0.80),a12=0.025 ,a13=0.033 ,a23=0.062,a123= -0.003,b12=0.01 ,b13=0.012 ,b23= 0.032,b123= -0.001,r11=c(2020,382,545,553,561,577,393,4969))

#7. Starting value:
list(pr=0.50, se=c(0.50,0.50,0.50),sp=c(0.50,0.50,0.50),a12=0.0 ,a13=0.0 ,a23=0.0,a123= 0.0,b12=0.0 ,b13=0.0 ,b23= 0.0,b123= 0.0,r11=c(2020,382,545,553,561,577,393,4969))

#8. Starting value:
list(pr=0.30, se=c(0.60,0.60,0.90),sp=c(0.90,0.90,0.90),a12=0.01 ,a13=0.0 ,a23=0.0,a123= 0.001,b12=0.001 ,b13=0.0 ,b23= 0.0,b123= 0.0,r11=c(2020,382,545,553,561,577,393,4969))

#9. Starting value:
list(pr=0.375, se=c(0.79,0.795,0.857),sp=c(0.914,0.910,0.951),a12=0.05 ,a13=0.05 ,a23=0.05,a123= 0.0,b12=0.003 ,b13=0.003 ,b23= 0.003,b123=0.00,r11=c(2020,382,545,553,561,577,393,4969))

#Simulation5:
list(r1=c(1186,20,253,141,113,61,549,7677),n1=10000)

#1. Starting value:
list(pr=0.20, se=c(0.72,0.65,0.97),sp=c(0.98,0.99,0.98),a12=0.129 ,a13=0.008 ,a23=0.012,a123= 0.0,b12=0.001 ,b13=0.003 ,b23= 0.001,b123=0.00,r11=c(1186,20,253,141,113,61,549,7677))

#2. Starting value:
list(pr=0.20, se=c(0.72,0.65,0.97),sp=c(0.98,0.99,0.98),a12=0.150 ,a13=0.009 ,a23=0.015,a123= 0.00,b12=0.002 ,b13=0.004 ,b23= 0.002,b123= 0.00,r11=c(1186,20,253,141,113,61,549,7677))

#3. Starting value:
list(pr=0.20, se=c(0.72,0.65,0.97),sp=c(0.98,0.99,0.98),a12=0.09 ,a13=0.003 ,a23=0.007,a123= 0.0,b12=0.001 ,b13=0.0 ,b23= 0.0,b123=0.00,r11=c(1186,20,253,141,113,61,549,7677))

#4. Starting value:
list(pr=0.15, se=c(0.77,0.70,0.98),sp=c(0.99,0.999,0.99),a12=0.116 ,a13=0.006 ,a23=0.022,a123= 0.0,b12=0.0003 ,b13=0.001 ,b23= 0.0003,b123= 0.0,r11=c(1186,20,253,141,113,61,549,7677))

#5. Starting value:
list(pr=0.20, se=c(0.65,0.65,0.95),sp=c(0.95,0.95,0.95),a12=0.137 ,a13=0.010 ,a23=0.0156,a123= 0.0,b12=0.005 ,b13=0.007 ,b23= 0.005,b123= 0.00,r11=c(1186,20,253,141,113,61,549,7677))

#6. Starting value:
list(pr=0.23, se=c(0.70,0.69,0.95),sp=c(0.99,0.99,0.99),a12=0.08 ,a13=0.0 ,a23=0.0,a123= 0.0,b12=0.001 ,b13=0.0 ,b23= 0.0,b123=0.0,r11=c(1186,20,253,141,113,61,549,7677))

#7. Starting value:
list(pr=0.50, se=c(0.50,0.50,0.50),sp=c(0.50,0.50,0.50),a12=0.0 ,a13=0.0 ,a23=0.0,a123= 0.0,b12=0.0 ,b13=0.0 ,b23= 0.0,b123= 0.0,r11=c(1186,20,253,141,113,61,549,7677))

#8. Starting value:
list(pr=0.30, se=c(0.85,0.80,0.70),sp=c(0.99,0.99,0.90),a12=0.0 ,a13=0.0 ,a23=0.08,a123= 0.0,b12=0.0 ,b13=0.0 ,b23= 0.001,b123= 0.0,r11=c(1186,20,253,141,113,61,549,7677))

#9. Starting value:
list(pr=0.159, se=c(0.916,0.830,0.984),sp=c(0.982,0.992,0.936),a12=0.05 ,a13=0.005 ,a23=0.049,a123= 0.0,b12=0.003 ,b13=0.003 ,b23= 0.003,b123=0.00,r11=c(1186,20,253,141,113,61,549,7677))
