# Supplementary material for: An Iterative, Frequentist Approach for Latent Class Analysis to Evaluate Conditionally Dependent Diagnostic Tests
Source: Front Vet Sci. 2021 Feb 10;8:588176. doi: 10.3389/fvets.2021.588176 (PMC7928357; doi:10.3389/fvets.2021.588176)
Supplement: Supplementary file 1 [file Data_Sheet_1.zip › Supplementary_Tables_S1_S26.docx]

**Supplementary Tables**

**Abbreviations:**

Sen 1 - Sen 3: The sensitivities of tests 1-3

Spe 1 - Spe 3: The specifities of tests 1-3

Prev: The prevalence of the disease in the population

std.: The standardized value of the dependency term

lCl: lower confidence limit

uCl: upper confidence limit

LL: The log-likelihood value for the chosen parameter values

E: Entropy

${}_{ij}^{+}$: The dependency of the sensitivities of test i and test j

${}_{ij}^{-}$: The dependency of the specifities of test i and test j

**Table S1: Well-chosen starting values for the stepwise latent class algorithm for all five simulation scenarios**

| **Parameter** | **Starting values 1** | **Starting values 2** | **Starting values 3** | **Starting values 4** | **Starting values 5** | **Starting values 6** |
| --- | --- | --- | --- | --- | --- | --- |
| **Three independent tests** | | | | | | |
| Prevalence | 0.300 | 0.300 | 0.300 | 0.350 | 0.400 | 0.400 |
| Sensitivity 1 | 0.900 | 0.900 | 0.900 | 0.900 | 0.920 | 0.920 |
| Sensitivity 2 | 0.850 | 0.850 | 0.850 | 0.900 | 0.830 | 0.830 |
| Sensitivity 3 | 0.900 | 0.900 | 0.900 | 0.900 | 0.940 | 0.940 |
| Specifity 1 | 0.950 | 0.950 | 0.950 | 0.950 | 0.920 | 0.920 |
| Specifity 2 | 0.950 | 0.950 | 0.950 | 0.950 | 0.930 | 0.930 |
| Specifity 3 | 0.990 | 0.990 | 0.990 | 0.950 | 0.990 | 0.990 |
| ${}_{12}^{+}$  (std.) | 0.000  (0.000) | 0.000 (0.000) | 0.010  (0.093) | 0.000 (0.000) | 0.000 (0.000) | 0.000 (0.000) |
| ${}_{13}^{+}$  (std.) | 0.000  (0.000) | 0.000  (0.000) | 0.010  (0.111) | 0.000 (0.000) | 0.000 (0.000) | 0.000  (0.000) |
| ${}_{23}^{+}$  (std.) | 0.000  (0.000) | 0.030  (0.280) | 0.010  (0.093) | 0.000 (0.000) | 0.000 (0.000) | 0.030  (0.466) |
| ${}_{123}^{+}$  (std.) | 0.000  (0.000) | 0.001  (0.031) | 0.000 (0.000) | 0.000 (0.000) | 0.000 (0.000) | 0.001  (0.041) |
| ${}_{12}^{-}$  (std.) | 0.000  (0.000) | 0.000 (0.000) | 0.003  (0.063) | 0.000 (0.000) | 0.000 (0.000) | 0.000 (0.000) |
| ${}_{13}^{-}$  (std.) | 0.000  (0.000) | 0.000  (0.000) | 0.003  (0.138) | 0.000 (0.000) | 0.000 (0.000) | 0.000  (0.000) |
| ${}_{23}^{-}$  (std.) | 0.000  (0.000) | 0.009  (0.350) | 0.003  (0.138) | 0.000 (0.000) | 0.000 (0.000) | 0.009  (0.355) |
| ${}_{123}^{-}$  (std.) | 0.000  (0.000) | 0.001  (0.100) | -0.003  (-0.635) | 0.000 (0.000) | 0.000 (0.000) | 0.001  (0.145) |
| **Two highly dependent tests with high prevalence** | | | | | | |
| Prevalence | 0.400 | 0.400 | 0.400 | 0.300 | 0.450 | 0.450 |
| Sensitivity 1 | 0.900 | 0.900 | 0.900 | 0.850 | 0.930 | 0.930 |
| Sensitivity 2 | 0.700 | 0.700 | 0.700 | 0.750 | 0.660 | 0.660 |
| Sensitivity 3 | 0.650 | 0.650 | 0.650 | 0.720 | 0.620 | 0.620 |
| Specifity 1 | 0.990 | 0.990 | 0.990 | 0.990 | 0.990 | 0.990 |
| Specifity 2 | 0.800 | 0.800 | 0.800 | 0.850 | 0.750 | 0.750 |
| Specifity 3 | 0.850 | 0.850 | 0.850 | 0.890 | 0.890 | 0.890 |
| ${}_{12}^{+}$  (std.) | 0.000  (0.000) | 0.000 (0.000) | 0.000 (0.000) | 0.000 (0.000) | 0.000 (0.000) | 0.000 (0.000) |
| ${}_{13}^{+}$  (std.) | 0.000  (0.000) | 0.000 (0.000) | 0.000 (0.000) | 0.000 (0.000) | 0.000 (0.000) | 0.000 (0.000) |
| ${}_{23}^{+}$  (std.) | 0.121  (0.600) | 0.140  (0.650) | 0.090  (0.411) | 0.117 (0.600) | 0.138 (0.600) | 0.110  (0.478) |
| ${}_{123}^{+}$  (std.) | 0.000  (0.000) | 0.000 (0.000) | 0.000 (0.000) | 0.000 (0.000) | 0.000 (0.000) | 0.000 (0.000) |
| ${}_{12}^{-}$  (std.) | 0.000  (0.000) | 0.000 (0.000) | 0.000 (0.000) | 0.000 (0.000) | 0.000 (0.000) | 0.000 (0.000) |
| ${}_{13}^{-}$  (std.) | 0.000  (0.000) | 0.000 (0.000) | 0.000 (0.000) | 0.000 (0.000) | 0.000 (0.000) | 0.000 (0.000) |
| ${}_{23}^{-}$  (std.) | 0.086  (0.600) | 0.095  (0.670) | 0.065  (0.455) | 0.067 (0.600) | 0.108 (0.600) | 0.095  (0.701) |
| ${}_{123}^{-}$  (std.) | 0.000  (0.000) | 0.000 (0.000) | 0.000 (0.000) | 0.000 (0.000) | 0.000 (0.000) | 0.000 (0.000) |
| **Two highly dependent tests with low prevalence** | | | | | | |
| Prevalence | 0.030 | 0.030 | 0.030 | 0.010 | 0.040 | 0.030 |
| Sensitivity 1 | 0.900 | 0.900 | 0.900 | 0.850 | 0.930 | 0.800 |
| Sensitivity 2 | 0.700 | 0.700 | 0.700 | 0.750 | 0.660 | 0.800 |
| Sensitivity 3 | 0.650 | 0.650 | 0.650 | 0.720 | 0.620 | 0.800 |
| Specifity 1 | 0.990 | 0.990 | 0.990 | 0.990 | 0.990 | 0.800 |
| Specifity 2 | 0.800 | 0.800 | 0.800 | 0.850 | 0.750 | 0.800 |
| Specifity 3 | 0.850 | 0.850 | 0.850 | 0.890 | 0.780 | 0.800 |
| ${}_{12}^{+}$  (std.) | 0.000  (0.000) | 0.000  (0.000) | 0.000  (0.000) | 0.000 (0.000) | 0.000 (0.000) | 0.000 (0.000) |
| ${}_{13}^{+}$  (std.) | 0.000  (0.000) | 0.000  (0.000) | 0.000  (0.000) | 0.000 (0.000) | 0.000 (0.000) | 0.000 (0.000) |
| ${}_{23}^{+}$  (std.) | 0.121  (0.600) | 0.140  (0.650) | 0.090  (0.411) | 0.117 (0.600) | 0.138 (0.600) | 0.110  (0.478) |
| ${}_{123}^{+}$  (std.) | 0.000  (0.000) | 0.000  (0.000) | 0.000  (0.000) | 0.000 (0.000) | 0.000 (0.000) | 0.000 (0.000) |
| ${}_{12}^{-}$  (std.) | 0.000  (0.000) | 0.000  (0.000) | 0.000  (0.000) | 0.000 (0.000) | 0.000 (0.000) | 0.000 (0.000) |
| ${}_{13}^{-}$  (std.) | 0.000  (0.000) | 0.000  (0.000) | 0.000  (0.000) | 0.000 (0.000) | 0.000 (0.000) | 0.000 (0.000) |
| ${}_{23}^{-}$  (std.) | 0.086  (0.600) | 0.095  (0.670) | 0.065  (0.455) | 0.067 (0.600) | 0.108 (0.600) | 0.095  (0.701) |
| ${}_{123}^{-}$  (std.) | 0.000  (0.000) | 0.000  (0.000) | 0.000  (0.000) | 0.000 (0.000) | 0.000 (0.000) | 0.000 (0.000) |
| **Three medium dependent tests with medium prevalence** | | | | | | |
| Prevalence | 0.400 | 0.400 | 0.400 | 0.350 | 0.320 | 0.400 |
| Sensitivity 1 | 0.800 | 0.800 | 0.800 | 0.850 | 0.600 | 0.800 |
| Sensitivity 2 | 0.660 | 0.660 | 0.660 | 0.730 | 0.600 | 0.800 |
| Sensitivity 3 | 0.700 | 0.700 | 0.700 | 0.750 | 0.600 | 0.800 |
| Specifity 1 | 0.950 | 0.950 | 0.950 | 0.980 | 0.800 | 0.800 |
| Specifity 2 | 0.850 | 0.850 | 0.850 | 0.880 | 0.800 | 0.800 |
| Specifity 3 | 0.880 | 0.880 | 0.880 | 0.920 | 0.800 | 0.800 |
| ${}_{12}^{+}$  (std.) | 0.038  (0.200) | 0.048  (0.253) | 0.025  (0.132) | 0.0317  (0.200) | 0.048  (0.200) | 0.025  (0.156) |
| ${}_{13}^{+}$  (std.) | 0.046  (0.250) | 0.055  (0.300) | 0.033  (0.180) | 0.0387  (0.250) | 0.060  (0.250) | 0.033  (0.200) |
| ${}_{23}^{+}$  (std.) | 0.087  (0.400) | 0.097  (0.450) | 0.062  (0.286) | 0.0769  (0.400) | 0.096  (0.400) | 0.062  (0.380) |
| ${}_{123}^{+}$  (std.) | -0.004  (-0.050) | -0.005  (-0.060) | -0.003  (-0.035) | -0.003  (-0.050) | -0.006  (-0.050) | -0.003  (-0.046) |
| ${}_{12}^{-}$  (std.) | 0.0156  (0.200) | 0.020  (0.260) | 0.010  (0.128) | 0.009  (0.200) | 0.032  (0.200) | 0.010  (0.063) |
| ${}_{13}^{-}$  (std.) | 0.018  (0.250) | 0.022  (0.311) | 0.012  (0.170) | 0.010  (0.250) | 0.040  (0.250) | 0.012  (0.075) |
| ${}_{23}^{-}$  (std.) | 0.046  (0.400) | 0.050  (0.450) | 0.032  (0.280) | 0.035  (0.400) | 0.064  (0.400) | 0.032  (0.200) |
| ${}_{123}^{-}$  (std.) | -0.0013  (-0.050) | -0.002  (-0.080) | -0.001  (-0.040) | -0.001  (-0.050) | -0.003  (-0.050) | -0.001  (-0.016) |
| **Brucellosis simulation** | | | | | | |
| Prevalence | 0.200 | 0.200 | 0.200 | 0.150 | 0.200 | 0.230 |
| Sensitivity 1 | 0.720 | 0.720 | 0.720 | 0.770 | 0.650 | 0.700 |
| Sensitivity 2 | 0.650 | 0.650 | 0.650 | 0.700 | 0.650 | 0.690 |
| Sensitivity 3 | 0.970 | 0.970 | 0.970 | 0.980 | 0.950 | 0.950 |
| Specifity 1 | 0.980 | 0.980 | 0.980 | 0.990 | 0.950 | 0.990 |
| Specifity 2 | 0.990 | 0.990 | 0.990 | 0.999 | 0.950 | 0.990 |
| Specifity 3 | 0.980 | 0.980 | 0.980 | 0.990 | 0.950 | 0.990 |
| ${}_{12}^{+}$  (std.) | 0.129 (0.600) | 0.150  (0.700) | 0.090  (0.420) | 0.116  (0.600) | 0.137  (0.600) | 0.080  (0.380) |
| ${}_{13}^{+}$  (std.) | 0.008  (0.100) | 0.009  (0.120) | 0.003  (0.040) | 0.006  (0.100) | 0.010  (0.100) | 0.000  (0.000) |
| ${}_{23}^{+}$  (std.) | 0.012  (0.150) | 0.015  (0.185) | 0.007  (0.086) | 0.022  (0.150) | 0.0156  (0.150) | 0.000  (0.000) |
| ${}_{123}^{+}$  (std.) | 0.000  (0.000) | 0.000  (0.000) | 0.000  (0.000) | 0.000  (0.000) | 0.000  (0.000) | 0.000  (0.000) |
| ${}_{12}^{-}$  (std.) | 0.001  (0.100) | 0.002  (0.150) | 0.001  (0.100) | 0.000^1^  (0.100) | 0.005  (0.100) | 0.001  (0.100) |
| ${}_{13}^{-}$  (std.) | 0.003  (0.150) | 0.004  (0.200) | 0.000  (0.000) | 0.001  (0.150) | 0.007  (0.150) | 0.000  (0.000) |
| ${}_{23}^{-}$  (std.) | 0.001  (0.100) | 0.002  (0.150) | 0.000  (0.000) | 0.000^1^  (0.100) | 0.005  (0.100) | 0.000  (0.000) |
| ${}_{123}^{-}$  (std.) | 0.000  (0.000) | 0.000  (0.000) | 0.000  (0.000) | 0.000  (0.000) | 0.000  (0.000) | 0.000  (0.000) |

^1^by rounding

**Table S2: Poorly chosen starting values for the stepwise latent class algorithm for all five simulation scenarios**

| **Parameter** | **Starting values 1** | **Starting values 2** | **Starting values 3** |
| --- | --- | --- | --- |
| **Three independent tests** | | | |
| Prevalence | 0.500 | 0.250 | 0.280 |
| Sensitivity 1 | 0.500 | 0.850 | 0.700 |
| Sensitivity 2 | 0.500 | 0.850 | 0.700 |
| Sensitivity 3 | 0.500 | 0.850 | 0.990 |
| Specifity 1 | 0.500 | 0.900 | 0.800 |
| Specifity 2 | 0.500 | 0.950 | 0.800 |
| Specifity 3 | 0.500 | 0.930 | 0.990 |
| ${}_{12}^{+}$(std.) | 0.000 (0.000) | 0.000 (0.000) | 0.040 (0.200) |
| ${}_{13}^{+}$(std.) | 0.000 (0.000) | 0.010 (0.080) | 0.000 (0.000) |
| ${}_{23}^{+}$(std.) | 0.000 (0.000) | 0.000 (0.000) | 0.000 (0.000) |
| ${}_{123}^{+}$(std.) | 0.000 (0.000) | 0.000 (0.000) | -0.001 (-0.050) |
| ${}_{12}^{-}$ (std.) | 0.000 (0.000) | 0.000 (0.000) | 0.009 (0.060) |
| ${}_{13}^{-}$ (std.) | 0.000 (0.000) | 0.001 (0.008) | 0.000 (0.000) |
| ${}_{23}^{-}$ (std.) | 0.000 (0.000) | 0.000 (0.000) | 0.000 (0.000) |
| ${}_{123}^{-}$(std.) | 0.000 (0.000) | 0.000 (0.000) | -0.001 (-0.063) |
| **Two highly dependent tests with high prevalence** | | | |
| Prevalence | 0.500 | 0.300 | 0.395 |
| Sensitivity 1 | 0.500 | 0.900 | 0.696 |
| Sensitivity 2 | 0.500 | 0.800 | 0.895 |
| Sensitivity 3 | 0.500 | 0.800 | 0.845 |
| Specifity 1 | 0.500 | 0.990 | 0.850 |
| Specifity 2 | 0.500 | 0.900 | 0.923 |
| Specifity 3 | 0.500 | 0.900 | 0.973 |
| ${}_{12}^{+}$(std.) | 0.000 (0.000) | 0.000 (0.000) | 0.010 (0.071) |
| ${}_{13}^{+}$(std.) | 0.000 (0.000) | 0.000 (0.000) | 0.010 (0.060) |
| ${}_{23}^{+}$(std.) | 0.000 (0.000) | 0.010 (0.063) | 0.100 (0.090) |
| ${}_{123}^{+}$(std.) | 0.000 (0.000) | 0.000 (0.000) | 0.000 (0.000) |
| ${}_{12}^{-}$ (std.) | 0.000 (0.000) | 0.000 (0.000) | 0.001 (0.011) |
| ${}_{13}^{-}$ (std.) | 0.000 (0.000) | 0.000 (0.000) | 0.001 (0.017) |
| ${}_{23}^{-}$ (std.) | 0.000 (0.000) | 0.001 (0.011) | 0.090 (2.010) |
| ${}_{123}^{-}$(std.) | 0.000 (0.000) | 0.000 (0.000) | 0.000 (0.000) |
| **Two highly dependent tests with low prevalence** | | | |
| Prevalence | 0.500 | 0.040 | 0.020 |
| Sensitivity 1 | 0.500 | 0.900 | 0.600 |
| Sensitivity 2 | 0.500 | 0.800 | 0.896 |
| Sensitivity 3 | 0.500 | 0.800 | 0.843 |
| Specifity 1 | 0.500 | 0.990 | 0.982 |
| Specifity 2 | 0.500 | 0.900 | 0.924 |
| Specifity 3 | 0.500 | 0.900 | 0.973 |
| ${}_{12}^{+}$(std.) | 0.000 (0.000) | 0.000 (0.000) | 0.010 (0.071) |
| ${}_{13}^{+}$(std.) | 0.000 (0.000) | 0.000 (0.000) | 0.010 (0.060) |
| ${}_{23}^{+}$(std.) | 0.000 (0.000) | 0.010 (0.063) | 0.100 (0.090) |
| ${}_{123}^{+}$(std.) | 0.000 (0.000) | 0.000 (0.000) | 0.000 (0.000) |
| ${}_{12}^{-}$ (std.) | 0.000 (0.000) | 0.000 (0.000) | 0.001 (0.011) |
| ${}_{13}^{-}$ (std.) | 0.000 (0.000) | 0.000 (0.000) | 0.001 (0.017) |
| ${}_{23}^{-}$ (std.) | 0.000 (0.000) | 0.001 (0.011) | 0.090 (2.010) |
| ${}_{123}^{-}$(std.) | 0.000 (0.000) | 0.000 (0.000) | 0.000 (0.000) |
| **Three medium dependent tests with medium prevalence** | | | |
| Prevalence | 0.500 | 0.300 | 0.375 |
| Sensitivity 1 | 0.500 | 0.600 | 0.790 |
| Sensitivity 2 | 0.500 | 0.600 | 0.795 |
| Sensitivity 3 | 0.500 | 0.800 | 0.857 |
| Specifity 1 | 0.500 | 0.900 | 0.914 |
| Specifity 2 | 0.500 | 0.900 | 0.910 |
| Specifity 3 | 0.500 | 0.900 | 0.951 |
| ${}_{12}^{+}$(std.) | 0.000 (0.000) | 0.010 (0.041) | 0.050 (0.300) |
| ${}_{13}^{+}$(std.) | 0.000 (0.000) | 0.000 (0.000) | 0.050 (0.350) |
| ${}_{23}^{+}$(std.) | 0.000 (0.000) | 0.000 (0.000) | 0.050 (0.354) |
| ${}_{123}^{+}$(std.) | 0.000 (0.000) | 0.001 (0.010) | 0.000 (0.000) |
| ${}_{12}^{-}$ (std.) | 0.000 (0.000) | 0.001 (0.011) | 0.030 (0.374) |
| ${}_{13}^{-}$ (std.) | 0.000 (0.000) | 0.000 (0.000) | 0.030 (0.500) |
| ${}_{23}^{-}$ (std.) | 0.000 (0.000) | 0.000 (0.000) | 0.030 (0.490) |
| ${}_{123}^{-}$(std.) | 0.000 (0.000) | 0.000 (0.000) | 0.000 (0.000) |
| **Brucellosis simulation** | | | |
| Prevalence | 0.500 | 0.300 | 0.159 |
| Sensitivity 1 | 0.500 | 0.850 | 0.916 |
| Sensitivity 2 | 0.500 | 0.800 | 0.830 |
| Sensitivity 3 | 0.500 | 0.700 | 0.984 |
| Specifity 1 | 0.500 | 0.990 | 0.982 |
| Specifity 2 | 0.500 | 0.990 | 0.992 |
| Specifity 3 | 0.500 | 0.900 | 0.936 |
| ${}_{12}^{+}$(std.) | 0.000 (0.000) | 0.000 (0.000) | 0.050 (0.500) |
| ${}_{13}^{+}$(std.) | 0.000 (0.000) | 0.000 (0.000) | 0.050 (1.500) |
| ${}_{23}^{+}$(std.) | 0.000 (0.000) | 0.080 (0.440) | 0.050 (1.000) |
| ${}_{123}^{+}$(std.) | 0.000 (0.000) | 0.000 (0.000) | 0.000 (0.000) |
| ${}_{12}^{-}$ (std.) | 0.000 (0.000) | 0.000 (0.000) | 0.003 (0.253) |
| ${}_{13}^{-}$ (std.) | 0.000 (0.000) | 0.000 (0.000) | 0.003 (0.092) |
| ${}_{23}^{-}$ (std.) | 0.000 (0.000) | 0.001 (0.034) | 0.003 (0.150) |
| ${}_{123}^{-}$(std.) | 0.000 (0.000) | 0.000 (0.000) | 0.000 (0.000) |

**Table S3: Results of the classical and the iterative LCA for the well-chosen starting values in the first simulation scenario**

| Para-meter | LCA for independent test | Starting values 1 | Starting values 2 | Starting values 3 | Starting values 4 | Starting values 5 | Starting values 6 |
| --- | --- | --- | --- | --- | --- | --- | --- |
| Prev  lCl  uCl | 0.299  0.271  0.380 | 0.300  0.272  0.390 | 0.301  0.273  0.330 | 0.311  0.282  0.339 | 0.299  0.270  0.327 | 0.300  0.272  0.329 | 0.300  0.271  0.328 |
| Sen 1  lCl  uCl | 0.910  0.883  0.90 | 0.898  0.879  0.960 | 0.930  0.914  0.946 | 0.875  0.854  0.895 | 0.905  0.887  0.923 | 0.898  0.879  0.916 | 0.937  0.922  0.952 |
| Sen 2  lCl  uCl | 0.853  0.831  0.885 | 0.850  0.828  0.882 | 0.827  0.803  0.851 | 0.827  0.804  0.850 | 0.858  0.837  0.880 | 0.850  0.828  0.872 | 0.823  0.799  0.846 |
| Sen 3  lCl  uCl | 0.889 0.869  0.908 | 0.892  0.873  0.920 | 0.853  0.831  0.875 | 0.868  0.847  0.889 | 0.889  0.869  0.908 | 0.892  0.874  0.912 | 0.856  0.834  0.878 |
| Spe 1  lCl  uCl | 0.950  0.937  0.964 | 0.949  0.936  0.963 | 0.965  0.953  0.976 | 0.952  0.934  0.965 | 0.950  0.937  0.964 | 0.949  0.935  0.963 | 0.966  0.954 0.977 |
| Spe 2  lCl  uCl | 0.960 0.947  0.982 | 0.959  0.947  0.971 | 0.950  0.937  0.964 | 0.961  0.949  0.973 | 0.960  0.948  0.973 | 0.959  0.947  0.971 | 0.947  0.933 0.961 |
| Spe 3  lCl  uCl | 0.979  0.971  0.988 | 0.981  0.973  0.990 | 0.966  0.954  0.977 | 0.984  0.976  0.992 | 0.978  0.969  0.987 | 0.982  0.973  0.990 | 0.965  0.954 0.976 |
| ${}_{12}^{+}$  (std.) | - | 0.003  (0.048) | -0.004  (-0.040) | 0.010  (0.078) | -0.008  (-0.081) | 0.003  (0.031) | -0.005  (-0.057) |
| ${}_{13}^{+}$  (std.) | - | 0.000^2^  (0.016) | 0.000^2^  (-0.002) | 0.007  (0.062) | -0.006  (-0.063) | 0.000^2^  (0.001) | -0.004  (-0.056) |
| ${}_{23}^{+}$  (std.) | - | 0.000^2^  (0.015) | 0.025  (0.188) | 0.007  (0.054) | -0.007  (-0.059) | 0.000^2^  (-0.001) | 0.024  (0.183) |
| ${}_{123}^{+}$  (std.) | - | -0.001  (-0.079) | 0.003  (0.099) | 0.002  (0.047) | 0.007  (0.218) | -0.001  (-0.031) | 0.005  (0.160) |
| ${}_{12}^{-}$  (std.) | - | 0.001  (0.002) | 0.000^2^  (0.006) | 0.004  (0.102) | 0.002  (0.041) | 0.001  (0.013) | 0.002  (0.041) |
| ${}_{13}^{-}$  (std.) | - | -0.001  (-0.040) | 0.002  (0.052) | 0.003  (0.124) | 0.003  (0.081) | -0.001  (-0.019) | 0.002  (0.052) |
| ${}_{23}^{-}$  (std.) | - | -0.001  (-0.041) | 0.009  (0.239) | 0.003  (0.134) | 0.002  (0.078) | -0.001  (-0.024) | 0.012  (0.287) |
| ${}_{123}^{-}$  (std.) | - | 0.000^2^  (0.166) | 0.000^2^  (0.010) | -0.003  (-0.737) | -0.003  (-0.470) | 0.000^2^  (0.045) | -0.001  (-0.167) |
| It.^1^ | - | 6 | 6 | 6 | 7 | 6 | 7 |
| LL | -1215.97 | -1215.97 | -1215.97 | -1215.97 | -1215.97 | -1215.97 | -1215.97 |
| AIC | 14.00 | 14.00 | 14.00 | 14.00 | 14.00 | 14.00 | 14.00 |
| BIC | 48.35 | 48.35 | 48.35 | 48.35 | 48.35 | 48.35 | 48.35 |
| E | 0.91 | 0.91 | 0.91 | 0.90 | 0.91 | 0.91 | 0.91 |

^1^ The number of the iterations the whole algorithm, not the ones of the EM algorithm performed at each step

^2^by rounding

**Table S4: Results of the classical and the iterative LCA for the well-chosen starting values in the second simulation scenario**

| Para-meter | LCA for independent test | Starting values 1 | Starting values 2 | Starting values 3 | Starting values 4 | Starting values 5 | Starting values 6 |
| --- | --- | --- | --- | --- | --- | --- | --- |
| Prev  lCl  uCl | 0.395  0.386  0.405 | 0.400  0.390  0.410 | 0.392  0.383  0.402 | 0.396  0.386  0.405 | 0.421  0.411  0.431 | 0.374  0.365  0.384 | 0.392  0.382  0.401 |
| Sen 1  lCl  uCl | 0.696  0.687  0.705 | 0.900  0.894  0.906 | 0.930  0.925  0.935 | 0.823  0.815  0.830 | 0.838  0.831  0.845 | 0.977  0.975  0.981 | 0.923  0.918  0.928 |
| Sen 2  lCl  uCl | 0.895  0.889  0.901 | 0.700  0.691  0.709 | 0.693  0.684  0.702 | 0.758  0.750  0.767 | 0.713  0.704  0.721 | 0.691  0.682  0.700 | 0.697  0.688  0.706 |
| Sen 3  lCl  uCl | 0.845  0.838  0.852 | 0.650  0.641  0.659 | 0.643  0.633  0.652 | 0.708  0.699  0.717 | 0.662  0.653  0.672 | 0.642  0.632  0.651 | 0.646  0.637  0.655 |
| Spe 1  lCl  uCl | 0.850  0.843  0.857 | 0.990  0.988  0.992 | 0.998  0.997  0.999 | 0.933  0.928  0.938 | 0.977  0.974  0.980 | 0.999  0.998  1.000 | 0.992  0.990  0.994 |
| Spe 2  lCl  uCl | 0.923  0.918  0.929 | 0.800  0.792  0.808 | 0.789  0.780  0.797 | 0.834  0.827  0.842 | 0.827  0.820  0.834 | 0.774  0.766  0.783 | 0.791  0.783  0.799 |
| Spe 3  lCl  uCl | 0.973  0.970  0.977 | 0.850  0.843  0.857 | 0.839  0.832  0.846 | 0.884  0.878  0.890 | 0.877  0.870  0.883 | 0.824  0.817  0.832 | 0.841  0.833  0.848 |
| ${}_{12}^{+}$  (std.) | - | 0.000  (0.000) | 0.001  (0.006) | 0.000^2^  (0.002) | -0.001  (-0.008) | 0.000^2^  (0.006) | 0.001  (0.010) |
| ${}_{13}^{+}$  (std.) | - | 0.000  (0.000) | 0.001  (0.004) | 0.000^2^  (0.002) | -0.001  (-0.006) | 0.000^2^  (0.004) | 0.001  (0.011) |
| ${}_{23}^{+}$  (std.) | - | 0.121  (0.600) | 0.125  (0.567) | 0.093  (0.477) | 0.116  (0.544) | 0.125  (0.564) | 0.121  (0.552) |
| ${}_{123}^{+}$  (std.) | - | 0.000  (0.000) | -0.001  (-0.014) | 0.000^2^  (-0.002) | 0.000^2^  (-0.002) | 0.000^2^  (-0.003) | 0.000^2^  (0.006) |
| ${}_{12}^{-}$  (std.) | - | 0.000  (0.000) | 0.000^2^  (-0.003) | -0.001  (-0.006) | 0.000^2^  (0.004) | 0.000^2^  (-0.083) | 0.000  (0.000) |
| ${}_{13}^{-}$  (std.) | - | 0.000  (0.000) | 0.000^2^  (-0.003) | 0.000^2^  (-0.006) | 0.000^2^  (0.005) | 0.000^2^  (-0.079) | 0.000^2^  (0.006) |
| ${}_{23}^{-}$  (std.) | - | 0.086  (0.600) | 0.091  (0.609) | 0.065  (0.547) | 0.069  (0.557) | 0.100  (0.627) | 0.092  (0.617) |
| ${}_{123}^{-}$  (std.) | - | 0.000  (0.000) | 0.000^2^  (0.024) | 0.000^2^  (0.006) | 0.000^2^  (-0.020) | 0.000^2^  (0.325) | 0.000^2^  (-0.029) |
| It.^1^ | - | 3 | 4 | 6 | 5 | 4 | 5 |
| LL | -15968.1 | -15968.1 | -15968.1 | -15968.1 | -15968.1 | -15968.1 | -15968.1 |
| AIC | 14.00 | 14.00 | 14.00 | 14.00 | 14.00 | 14.00 | 14.00 |
| BIC | 64.47 | 64.47 | 64.47 | 64.47 | 64.47 | 64.47 | 64.47 |
| E | 0.826 | 0.78 | 0.82 | 0.71 | 0.73 | 0.90 | 0.80 |

^1^ The number of the iterations the whole algorithm, not the ones of the EM algorithm performed at each step

^2^by rounding

**Table S5: Results of the classical and the iterative LCA for the well-chosen starting values in the third simulation scenario**

| Para-meter | LCA for independent test | Starting values 1 | Starting values 2 | Starting values 3 | Starting values 4 | Starting values 5 | Starting values 6 |
| --- | --- | --- | --- | --- | --- | --- | --- |
| Prev  lCl  uCl | 0.169  0.161  0.177 | 0.030  0.027  0.033 | 0.031  0.028  0.034 | 0.071  0.066  0.076 | 0.024  0.021  0.027 | 0.0002  0.0000  0.0006 | 0.029  0.025  0.032 |
| Sen 1  lCl  uCl | 0.130  0.124  0.137 | 0.900  0.894  0.906 | 0.912  0.906  0.918 | 0.328  0.319  0.337 | 0.859  0.852  0.866 | 0.036  0.032  0.040 | 0.888  0.882  0.894 |
| Sen 2  lCl  uCl | 0.896  0.890  0.902 | 0.701  0.692  0.710 | 0.672  0.662  0.681 | 0.799  0.791  0.807 | 0.836  0.828  0.843 | 0.670  0.661  0.679 | 0.726  0.717  0.735 |
| Sen 3  lCl  uCl | 0.843  0.835  0.850 | 0.650  0.640  0.659 | 0.621  0.612  0.631 | 0.748  0.739  0.756 | 0.766  0.758  0.774 | 1.000  NaN  NaN | 0.676  0.667  0.685 |
| Spe 1  lCl  uCl | 0.982  0.979  0.985 | 0.990  0.988  0.992 | 0.991  0.990  0.993 | 0.986  0.983  0.988 | 0.984  0.981  0.986 | 0.963  0.960  0.967 | 0.988  0.986  0.991 |
| Spe 2  lCl  uCl | 0.924  0.919  0.929 | 0.800  0.792  0.808 | 0.800  0.792  0.808 | 0.830  0.822  0.837 | 0.800  0.792  0.808 | 0.785  0.777  0.793 | 0.800  0.792  0.808 |
| Spe 3  lCl  uCl | 0.973  0.970  0.976 | 0.850  0.843  0.857 | 0.850  0.843  0.857 | 0.880  0.873  0.886 | 0.850  0.843  0.857 | 0.835  0.828  0.843 | 0.850  0.667  0.685 |
| ${}_{12}^{+}$  (std.) | - | 0.000  (0.000) | 0.001  (0.005) | -0.005  (-0.027) | -0.009  (-0.066) | -0.078  (-0.892) | 0.001  (0.010) |
| ${}_{13}^{+}$  (std.) | - | 0.000  (0.000) | 0.000^2^  (-0.001) | -0.005  (-0.024) | -0.018  (-0.120) | -0.049  (NaN) | 0.000  (0.000) |
| ${}_{23}^{+}$  (std.) | - | 0.121  (0.600) | 0.129  (0.565) | 0.085  (0.487) | 0.104  (0.665) | -0.506  (NaN) | 0.102  (0.490) |
| ${}_{123}^{+}$  (std.) | - | 0.000  (0.000) | 0.002  (0.037) | -0.001  (-0.014) | 0.002  (0.036) | 0.099  (NaN) | 0.003  (0.052) |
| ${}_{12}^{-}$  (std.) | - | 0.000  (0.000) | 0.000^2^  (0.003) | 0.000^2^  (0.007) | 0.001  (0.013) | 0.013  (0.169) | 0.000^2^  (0.002) |
| ${}_{13}^{-}$  (std.) | - | 0.000  (0.000) | 0.000^2^  (0.003) | 0.000^2^  (0.006) | 0.001  (0.027) | 0.013  (0.186) | 0.000^2^  (0.002) |
| ${}_{23}^{-}$  (std.) | - | 0.086  (0.600) | 0.086  (0.601) | 0.067  (0.545) | 0.084  (0.590) | 0.094  (0.617) | 0.086  (0.601) |
| ${}_{123}^{-}$  (std.) | - | 0.000  (0.000) | 0.000^2^  (-0.008) | 0.000^2^  (-0.008) | 0.000  (0.000) | -0.007  (-0.245) | 0.000^2^  (-0.008) |
| It.^1^ | - | 4 | 5 | 9 | 6 | 4 | 5 |
| LL | -9522.50 | -9522.50 | -9522.50 | -9522.50 | -9522.50 | -9522.50 | -9522.50 |
| AIC | 14.00 | 14.00 | 14.00 | 14.00 | 14.00 | 14.00 | 14.00 |
| BIC | 64.47 | 64.47 | 64.47 | 64.47 | 64.47 | 64.47 | 64.47 |
| E | 0.80 | 0.74 | 0.74 | 0.64 | 0.78 | 0.61 | 0.74 |

^1^ The number of the iterations the whole algorithm, not the ones of the EM algorithm performed at each step

^2^by rounding

**Table S6: Results of the classical and the iterative LCA for the well-chosen starting values in the fourth simulation scenario**

| Para-meter | LCA for independent test | Starting values 1 | Starting values 2 | Starting values 3 | Starting values 4 | Starting values 5 | Starting values 6 |
| --- | --- | --- | --- | --- | --- | --- | --- |
| Prev  lCl  uCl | 0.375  0.374  0.384 | 0.400  0.390  0.410 | 0.402  0.392  0.411 | 0.402  0.393  0.412 | 0.395  0.385  0.405 | 0.345  0.336 0.355 | 0.380  0.370  0.390 |
| Sen 1  lCl  uCl | 0.790  0.782  0.798 | 0.800  0.792  0.808 | 0.793  0.785  0.801 | 0.791  0.783  0.800 | 0.844  0.837  0.850 | 0.741  0.732 0.749 | 0.805  0.797  0.813 |
| Sen 2  lCl  uCl | 0.794  0.787  0.803 | 0.660  0.651  0.669 | 0.642  0.633  0.652 | 0.694  0.685  0.703 | 0.666  0.656  0.675 | 0.711  0.702 0.720 | 0.716  0.707  0.725 |
| Sen3  lCl  uCl | 0.857  0.850  0.864 | 0.700  0.690  0.710 | 0.684  0.675  0.694 | 0.733  0.724  0.742 | 0.706  0.697  0.715 | 0.737  0.728 0.746 | 0.759  0.750  0.768 |
| Spe 1  lCl  uCl | 0.914  0.908  0.919 | 0.950  0.946  0.954 | 0.947  0.942  0.952 | 0.950  0.943  0.952 | 0.972  0.969  0.975 | 0.856  0.849 0.863 | 0.929  0.923  0.934 |
| Spe 2  lCl  uCl | 0.910  0.905  0.916 | 0.850  0.843  0.857 | 0.840  0.832  0.847 | 0.875  0.868  0.881 | 0.849  0.842  0.856 | 0.834  0.827 0.842 | 0.868  0.861  0.874 |
| Spe 3  lCl  uCl | 0.951  0.944  0.955 | 0.880  0.874  0.886 | 0.871  0.865  0.878 | 0.905  0.899  0.910 | 0.880  0.873  0.886 | 0.851  0.844 0.858 | 0.898  0.892  0.904 |
| ${}_{12}^{+}$  (std.) | - | 0.038  (0.200) | 0.047  (0.242) | 0.024  (0.130) | 0.026  (0.154) | 0.055  (0.277) | 0.023  (0.127) |
| ${}_{13}^{+}$  (std.) | - | 0.046  (0.250) | 0.054  (0.287) | 0.033  (0.182) | 0.034  (0.201) | 0.069  (0.357) | 0.031  (0.185) |
| ${}_{23}^{+}$  (std.) | - | 0.087  (0.400) | 0.097  (0.435) | 0.066  (0.330) | 0.084  (0.400) | 0.078  (0.390) | 0.058  (0.302) |
| ${}_{123}^{+}$  (std.) | - | -0.004  (-0.050) | -0.005  (-0.051) | -0.002  (-0.022) | -0.005  (-0.063) | -0.004  (-0.045) | -0.004  (-0.050) |
| ${}_{12}^{-}$  (std.) | - | 0.016  (0.200) | 0.020  (0.238) | 0.009  (0.127) | 0.009  (0.154) | 0.036  (0.277) | 0.011  (0.126) |
| ${}_{13}^{-}$  (std.) | - | 0.018  (0.250) | 0.021  (0.285) | 0.012  (0.178) | 0.009  (0.173) | 0.046  (0.369) | 0.013  (0.164) |
| ${}_{23}^{-}$  (std.) | - | 0.046  (0.400) | 0.051  (0.411) | 0.033  (0.338) | 0.047  (0.400) | 0.052  (0.394) | 0.034  (0.330) |
| ${}_{123}^{-}$  (std.) | - | -0.001  (-0.050) | -0.001  (-0.019) | -0.001  (-0.051) | -0.001  (-0.021) | -0.004  (-0.091) | -0.001  (-0.032) |
| It.^1^ | - | 3 | 6 | 5 | 6 | 7 | 4 |
| LL | -15673.82 | -15673.8 | -15673.8 | -15673.8 | -15673.8 | -15673.8 | -15673.8 |
| AIC | 14.00 | 14.00 | 14.00 | 14.00 | 14.00 | 14.00 | 14.00 |
| BIC | 64.47 | 64.47 | 64.47 | 64.47 | 64.47 | 64.47 | 64.47 |
| E | 0.80 | 0.72 | 0.70 | 0.74 | 0.76 | 0.67 | 0.80 |

^1^ The number of the iterations the whole algorithm, not the ones of the EM algorithm performed at each step

**Table S7: Results of the classical and the iterative LCA for the well-chosen starting values in the fifth simulation scenario**

| Para-meter | LCA for independent test | Starting values 1 | Starting values 2 | Starting values 3 | Starting values 4 | Starting values 5 | Starting values 6 |
| --- | --- | --- | --- | --- | --- | --- | --- |
| Prev  lCl  uCl | 0159  0.151  0.166 | 0.200  0.192  0.208 | 0.208  0.200  0.216 | 0.187  0.179  0.195 | 0.204  0.196  0.211 | 0.186  0.178  0.194 | 0.180  0.173  0.188 |
| Sen 1  lCl  uCl | 0.916  0.910  0.921 | 0.720  0.711  0.729 | 0.691  0.682  0.700 | 0.781  0.773  0.789 | 0.729  0.720  0.737 | 0.748  0.740  0.757 | 0.801  0.793  0.809 |
| Sen 2  lCl  uCl | 0.830  0.822  0.837 | 0.650  0.641  0.659 | 0.622  0.613  0.632 | 0.706  0.700  0.714 | 0.645  0.635  0.654 | 0.684  0.675  0.693 | 0.725  0.716  0.733 |
| Sen3  lCl  uCl | 0.984  0.982  0.987 | 0.970  0.967  0.973 | 0.964  0.960  0.968 | 0.973  0.970  0.976 | 0.953  0.948  0.957 | 0.961  0.958  0.965 | 0.991  0.990  0.993 |
| Spe 1  lCl  uCl | 0.982  0.979  0.985 | 0.980  0.977  0.983 | 0.979  0.977  0.982 | 0.983  0.980  0.986 | 0.986  0.983  0.988 | 0.974  0.971  0.978 | 0.981  0.979  0.984 |
| Spe 2  lCl  uCl | 0.992  0.990  0.994 | 0.990  0.988  0.992 | 0.989  0.987  0.991 | 0.992  0.990  0.994 | 0.992  0.990  0.994 | 0.987  0.985  0.989 | 0.991  0.989  0.993 |
| Spe 3  lCl  uCl | 0.935  0.930  0.940 | 0.980  0.977  0.983 | 0.988  0.986  0.990 | 0.965  0.962  0.969 | 0.980  0.977  0.983 | 0.962  0.958  0.965 | 0.962  0.958  0.966 |
| ${}_{12}^{+}$  (std.) | - | 0.129  (0.600) | 0.145  (0.649) | 0.094  (0.500) | 0.121  (0.568) | 0.127  (0.628) | 0.084  (0.470) |
| ${}_{13}^{+}$  (std.) | - | 0.008  (0.100) | 0.011  (0.126) | 0.005  (0.071) | 0.007  (0.072) | 0.015  (0.176) | 0.002  (0.042) |
| ${}_{23}^{+}$  (std.) | - | 0.012  (0.150) | 0.017  (0.191) | 0.009  (0.120) | 0.023  (0.227) | 0.016  (0.175) | 0.002  (0.036) |
| ${}_{123}^{+}$  (std.) | - | 0.000  (0.000) | -0.001  (-0.028) | -0.002  (-0.015) | 0.000^2^  (-0.021) | 0.0001  (0.018) | -0.002  (-0.110) |
| ${}_{12}^{-}$  (std.) | - | 0.001  (0.100) | 0.001  (0.072) | 0.000  (0.000) | 0.000^2^  (0.020) | 0.002  (0.101) | 0.001  (0.058) |
| ${}_{13}^{-}$  (std.) | - | 0.003  (0.150) | 0.004  (0.241) | 0.000  (0.000) | 0.000^2^  (0.064) | 0.008  (0.263) | 0.000^2^  (-0.015) |
| ${}_{23}^{-}$  (std.) | - | 0.001  (0.100) | 0.002  (0.164) | 0.000  (0.000) | 0.000^2^  (0.001) | 0.005  (0.234) | 0.000^2^  (-0.020) |
| ${}_{123}^{-}$  (std.) | - | 0.000  (0.000) | 0.001  (0.312) | 0.000  (0.000) | 0.000^2^  (0.169) | 0.000^2^  (0.038) | 0.000^2^  (0.169) |
| It.^1^ | - | 3 | 6 | 7 | 6 | 7 | 6 |
| LL | -8624.43 | -8624.43 | -8624.43 | -8624.43 | -8624.43 | -8624.43 | -8624.43 |
| AIC | 14.00 | 14.00 | 14.00 | 14.00 | 14.00 | 14.00 | 14.00 |
| BIC | 64.47 | 64.47 | 64.47 | 64.47 | 64.47 | 64.47 | 64.47 |
| E | 0.95 | 0.92 | 0.92 | 0.91 | 0.91 | 0.91 | 0.93 |

^1^ The number of the iterations the whole algorithm, not the ones of the EM algorithm performed at each step

^2^by rounding

**Table S8: Results of the iterative LCA for the poorly chosen starting values in the first simulation scenario**

| Parameter | Starting values 1 | Starting values 2 | Starting values 3 |
| --- | --- | --- | --- |
| Prevalence  (lCl,uCl) | 0.500  (0.469,0.531) | 0.302  (0.273,0.330) | 0.304  (0.275,0.332) |
| Sen 1  (lCl,uCl) | 0.305  (0.276, 0.334 | 0.893  (0.874,0.912) | 0.862  (0.840,0.883) |
| Sen 2  (lCl,uCl) | 0.284  (0.256,0.312) | 0.859  (0.837,0.880) | 0.816  (0.791,0.840) |
| Sen 3  (lCl,uCl) | 0.281  (0.253,0.309) | 0.880  (0.860,0.900) | 0.922  (0.906,0.939) |
| Spe 1  (lCl,uCl) | 0.695  (0.666,0.724) | 0.949  (0.935,0.963) | 0.938  (0.923,0.953) |
| Spe 2  (lCl,uCl) | 0.716  (0.688,0.744) | 0.964  (0.953,0.976) | 0.948  (0.934,0.962) |
| Spe 3  (lCl,uCl) | 0.719  (0.691,0.747) | 0.978  (0.969,0.987) | 0.999  (0.996,1.000) |
| ${}_{12}^{+}$(std.) | 0.145 (0.700) | -0.005 (-0.055) | 0.029 (0.219) |
| ${}_{13}^{+}$(std.) | 0.155 (0.750) | 0.004 (0.041) | -0.003 (-0.032) |
| ${}_{23}^{+}$(std.) | 0.148 (0.731) | -0.006 (-0.049) | -0.003 (-0.030) |
| ${}_{123}^{+}$(std.) | 0.051 (0.541) | 0.006 (0.160) | 0.002 (0.047) |
| ${}_{12}^{-}$ (std.) | 0.145 (0.700) | 0.002 (0.041) | 0.011 (0.200) |
| ${}_{13}^{-}$ (std.) | 0.155 (0.750) | 0.003 (0.086) | 0.001 (0.096) |
| ${}_{23}^{-}$ (std.) | 0.148 (0.731) | 0.002 (0.063) | 0.001 (0.095) |
| ${}_{123}^{-}$(std.) | -0.051 (-0.541) | -0.002 (-0.385) | -0.001 (-0.715) |
| Iterations^1^ | 3 | 7 | 6 |
| LL | -1215.97 | -1215.97 | -1215.97 |
| AIC | 14.00 | 14.00 | 14.00 |
| BIC | 48.35 | 48.35 | 48.35 |
| E | 0.00 | 0.91 | 0.93 |

^1^ The number of the iterations the whole algorithm, not the ones of the EM algorithm performed at each step

**Table S9: Results of the iterative LCA for the poorly chosen starting values in the second simulation scenario**

| Parameter | Starting values 1 | Starting values 2 | Starting values 3 |
| --- | --- | --- | --- |
| Prevalence  (lCl,uCl) | 0.500  (0.490,0.510) | 0.403  (0.394,0.413) | 0.358  (0.349,0.367) |
| Sen 1  (lCl,uCl) | 0.366  (0.357, 0.375 | 0.695  (0.686,0.704) | 0.900  (0.895,0.907) |
| Sen 2  (lCl,uCl) | 0.400  (0.390,0.410) | 0.880  (0.874,0.886) | 0.746  (0.737,0.754) |
| Sen 3  (lCl,uCl) | 0.350  (0.341,0.359) | 0.828  (0.821,0.836) | 0.696  (0.687,0.705) |
| Spe 1  (lCl,uCl) | 0.634  (0.625,0.644) | 0.856  (0.849,0.863) | 0.932  (0.927,0.937) |
| Spe 2  (lCl,uCl) | 0.600  (0.590,0.610) | 0.925  (0.919,0.930) | 0.792  (0.785,0.801) |
| Spe 3  (lCl,uCl) | 0.650  (0.641,0.659) | 0.973  (0.970,0.977) | 0.843  (0.836,0.850) |
| ${}_{12}^{+}$(std.) | 0.107 (0.453) | 0.002 (0.015) | 0.009 (0.065) |
| ${}_{13}^{+}$(std.) | 0.107 (0.465) | 0.002 (0.015) | 0.009 (0.062) |
| ${}_{23}^{+}$(std.) | 0.160 (0.685) | 0.012 (0.098) | 0.096 (0.481) |
| ${}_{123}^{+}$(std.) | 0.018 (0.162) | -0.001 (-0.020) | 0.001 (0.012) |
| ${}_{12}^{-}$ (std.) | 0.107 (0.453) | 0.000^2^ (-0.013) | 0.001 (0.009) |
| ${}_{13}^{-}$ (std.) | 0.107 (0.465) | 0.000^2^ (-0.015) | 0.001 (0.008) |
| ${}_{23}^{-}$ (std.) | 0.160 (0.685) | 0.000^2^ (-0.002) | 0.091 (0.621) |
| ${}_{123}^{-}$(std.) | -0.018 (-0.163) | 0.001(0.075) | 0.000^2^ (0.025) |
| Iterations^1^ | 3 | 7 | 6 |
| LL | -15968.05 | -15968.05 | -15968.05 |
| AIC | 14.00 | 14.00 | 14.00 |
| BIC | 64.47 | 64.47 | 64.47 |
| E | 0.00 | 0.82 | 0.71 |

^1^ The number of the iterations the whole algorithm, not the ones of the EM algorithm performed at each step

^2^by rounding

**Table S10: Results of the iterative LCA for the poorly chosen starting values in the third simulation scenario**

| Parameter | Starting values 1 | Starting values 2 | Starting values 3 |
| --- | --- | --- | --- |
| Prevalence  (lCl,uCl) | 0.500  (0.490,0.510) | 0.158  (0.150,0.165) | 0.000  (0.000,0.010) |
| Sen 1  (lCl,uCl) | 0.037  (0.033,0.040) | 0.137  (0.130,0.143) | 0.063  (0.059,0.068) |
| Sen 2  (lCl,uCl) | 0.215  (0.207,0.223) | 0.913  (0.908,0.919) | 0.364  (0.354,0.373) |
| Sen 3  (lCl,uCl) | 0.165  (0.158,0.172) | 0.866  (0.860,0.873) | 0.655  (0.646,0.665) |
| Spe 1  (lCl,uCl) | 0.963  (0.960,0.967) | 0.982  (0.979,0.985) | 0.962  (0.959,0.966) |
| Spe 2  (lCl,uCl) | 0.785  (0.777,0.793) | 0.916  (0.910,0.921) | 0.780  (0.771,0.788) |
| Spe 3  (lCl,uCl) | 0.835  (0.828,0.842) | 0.966  (0.963,0.970) | 0.817  (0.809,0.825) |
| ${}_{12}^{+}$(std.) | 0.013 (0.168) | -0.004 (-0.043) | 0.024 (0.201) |
| ${}_{13}^{+}$(std.) | 0.013 (0.185) | -0.004 (-0.038) | -0.029 (-0.253) |
| ${}_{23}^{+}$(std.) | 0.094 (0.617) | 0.006 (0.056) | 0.056 (0.247) |
| ${}_{123}^{+}$(std.) | 0.007 (0.245) | 0.000^2^ (0.001) | -0.002 (-0.513) |
| ${}_{12}^{-}$ (std.) | 0.013 (0.168) | 0.001 (0.022) | 0.014 (0.171) |
| ${}_{13}^{-}$ (std.) | 0.013 (0.185) | 0.001 (0.024) | 0.012 (0.161) |
| ${}_{23}^{-}$ (std.) | 0.094 (0.617) | 0.002 (0.037) | 0.095 (0.594) |
| ${}_{123}^{-}$(std.) | -0.007 (-0.245) | -0.001 (-0.109) | -0.007 (-0.218) |
| Iterations^1^ | 3 | 9 | 9 |
| LL | -9522.50 | -9522.50 | -9522.50 |
| AIC | 14.00 | 14.00 | 14.00 |
| BIC | 64.47 | 64.47 | 64.47 |
| E | 0.00 | 0.811 | 0.01 |

^1^ The number of the iterations the whole algorithm, not the ones of the EM algorithm performed at each step

^2^by rounding

**Table S11: Results of the iterative LCA for the poorly chosen starting values in the fourth simulation scenario**

| Parameter | Starting values 1 | Starting values 2 | Starting values 3 |
| --- | --- | --- | --- |
| Prevalence  (lCl,uCl) | 0.500  (0.490,0.510) | 0.386  (0.377,0.397) | 0.053  (0.049,0.058) |
| Sen 1  (lCl,uCl) | 0.350  (0.340,0.360) | 0.770  (0.762,0.778) | 1.000  (NaN,NaN) |
| Sen 2  (lCl,uCl) | 0.354  (0.345,0.363) | 0.774  (0.766,0.783) | 0.849  (0.842,0.856) |
| Sen 3  (lCl,uCl) | 0.352  (0.343,0.361) | 0.856  (0.849,0.863) | 0.202  (194,0.210) |
| Spe 1  (lCl,uCl) | 0.650  (0.64,0.660) | 0.914  (0.909,0.919) | 0.688  (0.679,0.697) |
| Spe 2  (lCl,uCl) | 0.646  (0.637,0.655) | 0.910  (0.905,0.916) | 0.674  (0.665,0.683) |
| Spe 3  (lCl,uCl) | 0.648  (0.639,0.657) | 0.969  (0.962,0.969) | 0.640  (0.630,0.650) |
| ${}_{12}^{+}$(std.) | 0.116 (0.510) | 0.013 (0.077) | -0.161 (NaN) |
| ${}_{13}^{+}$(std.) | 0.133 (0.585) | 0.002 (0.014) | -0.192 (NaN) |
| ${}_{23}^{+}$(std.) | 0.134 (0.585) | 0.002 (0.012) | -0.179 (-1.246) |
| ${}_{123}^{+}$(std.) | 0.024 (0.216) | 0.000^2^ (-0.003) | 0.154 (NaN) |
| ${}_{12}^{-}$ (std.) | 0.116 (0.510) | 0.000^2^ (0.002) | 0.112 (0.515) |
| ${}_{13}^{-}$ (std.) | 0.133 (0.585) | -0.001 (-0.020) | 0.158 (0.709) |
| ${}_{23}^{-}$ (std.) | 0.134 (0.585) | -0.001 (-0.020) | 0.156 (0.691) |
| ${}_{123}^{-}$(std.) | -0.024 (-0.216) | 0.012 (0.082) | -0.039 (-0.378) |
| Iterations^1^ | 3 | 7 | 6 |
| LL | -15673.82 | -15673.82 | -15673.82 |
| AIC | 14.00 | 14.00 | 14.00 |
| BIC | 64.47 | 64.47 | 64.47 |
| E | 0.00 | 0.80 | 0.00 |

^1^ The number of the iterations the whole algorithm, not the ones of the EM algorithm performed at each step

^2^by rounding

**Table S12: Results of the iterative LCA for the poorly chosen starting values in the fifth simulation scenario**

| Parameter | Starting values 1 | Starting values 2 | Starting values 3 |
| --- | --- | --- | --- |
| Prevalence  (lCl,uCl) | 0.500  (0.490,0.510) | 0.173  (0.166,0.180) | 0.000  (NaN,NaN) |
| Sen 1  (lCl,uCl) | 0.160  (0.153,0.167) | 0.924  (0.919,0.929) | 0.473  (0.463,0.483) |
| Sen 2  (lCl,uCl) | 0.138  (0.131,0.145) | 0.755  (0.746,0.762) | 0.786  (0.778,0.794) |
| Sen 3  (lCl,uCl) | 0.210  (0.202,0.218) | 0.900  (0.894,0.906) | 0.670  (0.660,0.679) |
| Spe 1  (lCl,uCl) | 0.840  (0.833,0.847) | 1.000  (0.999,1.000) | 0.840  (0.833,0.847) |
| Spe 2  (lCl,uCl) | 0.862  (0.855,0.869) | 0.991  (0.989,883) | 0.862  (0.855,0.869) |
| Spe 3  (lCl,uCl) | 0.790  (0.782,0.798) | 0.934  (0.929,0.939) | 0.790  (0.782,0.798) |
| ${}_{12}^{+}$(std.) | 0.098 (0.779) | 0.000^2^ (-0.002) | 0.174 (0.853) |
| ${}_{13}^{+}$(std.) | 0.110 (0.738) | 0.000^2^ (0.003) | 0.121 (0.516) |
| ${}_{23}^{+}$(std.) | 0.100 (0.718) | 0.064 (0.495) | 0.410 (2.126) |
| ${}_{123}^{+}$(std.) | 0.0612 (1.202) | -0.001 (-0.027) | -0.103 (-1.070) |
| ${}_{12}^{-}$ (std.) | 0.098 (0.779) | 0.000^2^ (0.025) | 0.098 (0.779) |
| ${}_{13}^{-}$ (std.) | 0.110 (0.738) | 0.000^2^ (0.013) | 0.110 (0.738) |
| ${}_{23}^{-}$ (std.) | 0.100 (0.718) | 0.001 (0.044) | 0.101 (0.718) |
| ${}_{123}^{-}$(std.) | -0.062 (-1.202) | 0.000^2^ (-0.147) | -0.062 (-1.202) |
| Iterations^1^ | 3 | 5 | 5 |
| LL | -8624.429 | -8624.429 | -8624.429 |
| AIC | 14.00 | 14.00 | 14.00 |
| BIC | 64.47 | 64.47 | 64.47 |
| E | 0.00 | 0.94 | 0.63 |

^1^ The number of the iterations the whole algorithm, not the ones of the EM algorithm performed at each step

^2^by rounding

**Table S13: Results of the classical and the iterative LCA for the well-chosen starting values in the third simulation scenario with only positive pairwise dependencies allowed**

| Para-meter | LCA for independent test | Starting values 1 | Starting values 2 | Starting values 3 | Starting values 4 | Starting values 5 | Starting values 6 |
| --- | --- | --- | --- | --- | --- | --- | --- |
| Prev  lCl  uCl | 0.169  0.161  0.177 | 0.030  0.027  0.033 | 0.031  0.028  0.034 | 0.071  0.066  0.076 | 0.024  0.021  0.027 | 0.028  0.025  0.031 | 0.029  0.025  0.032 |
| Sen 1  lCl  uCl | 0.130  0.124  0.137 | 0.900  0.894  0.906 | 0.912  0.906  0.918 | 0.325  0.316  0.334 | 0.857  0.850  0.864 | 0.958  0.953  0.962 | 0.888  0.882  0.894 |
| Sen 2  lCl  uCl | 0.896  0.890  0.902 | 0.701  0.692  0.710 | 0.672  0.662  0.681 | 0.796  0.788  0.804 | 0.832  0.824  0.839 | 0.688  0.679  0.697 | 0.726  0.717  0.735 |
| Sen 3  lCl  uCl | 0.843  0.835  0.850 | 0.650  0.640  0.659 | 0.621  0.612  0.631 | 0.745  0.737  0.753 | 0.756  0.749  0.766 | 0.640  0.630  0.649 | 0.676  0.667  0.685 |
| Spe 1  lCl  uCl | 0.982  0.979  0.985 | 0.990  0.988  0.992 | 0.991  0.990  0.993 | 0.986  0.983  0.988 | 0.983  0.980  0.986 | 0.990  0.988  0.992 | 0.988  0.986  0.991 |
| Spe 2  lCl  uCl | 0.924  0.919  0.929 | 0.800  0.792  0.808 | 0.800  0.792  0.808 | 0.830  0.822  0.837 | 0.800  0.792  0.808 | 0.800  0.790  0.810 | 0.800  0.792  0.808 |
| Spe 3  lCl  uCl | 0.973  0.970  0.976 | 0.850  0.843  0.857 | 0.850  0.843  0.857 | 0.880  0.873  0.886 | 0.850  0.843  0.857 | 0.849  0.842  0.856 | 0.850  0.667  0.685 |
| ${}_{12}^{+}$  (std.) | - | 0.000  (0.000) | 0.000  (0.005) | 0.000  (0.000) | 0.001  (0.004) | 0.000^2^  (0.002) | 0.001  (0.010) |
| ${}_{13}^{+}$  (std.) | - | 0.000  (0.000) | 0.000  (0.000) | 0.000  (0.000) | 0.000  (0.000) | 0.000^2^  (0.002) | 0.000  (0.000) |
| ${}_{23}^{+}$  (std.) | - | 0.121  (0.600) | 0.129  (0.565) | 0.082  (0.464) | 0.102  (0.637) | 0.111  (0.500) | 0.102  (0.490) |
| ${}_{123}^{+}$  (std.) | - | 0.000  (0.000) | 0.002  (0.037) | -0.001  (-0.004) | 0.004  (0.064) | 0.009  (0.196) | 0.003  (0.052) |
| ${}_{12}^{-}$  (std.) | - | 0.000  (0.000) | 0.000^2^  (0.003) | 0.000^2^  (0.003) | 0.001  (0.013) | 0.000^2^  (0.010) | 0.000^2^  (0.002) |
| ${}_{13}^{-}$  (std.) | - | 0.000  (0.000) | 0.000^2^  (0.003) | 0.000^2^  (0.003) | 0.001  (0.024) | 0.000^2^  (0.007) | 0.000^2^  (0.002) |
| ${}_{23}^{-}$  (std.) | - | 0.086  (0.600) | 0.086  (0.601) | 0.067  (0.548) | 0.085  (0.590) | 0.087  (0.604) | 0.086  (0.601) |
| ${}_{123}^{-}$  (std.) | - | 0.000  (0.000) | 0.000^2^  (-0.008) | 0.000^2^  (0.009) | 0.000^2^  (0.024) | 0.000^2^  (-0.023) | 0.000^2^  (-0.008) |
| Iterations^1^ | - | 4 | 5 | 9 | 7 | 5 | 7 |
| LL | -9522.50 | -9522.50 | -9522.50 | -9522.50 | -9522.50 | -9522.50 | -9522.50 |
| AIC | 14.00 | 14.00 | 14.00 | 14.00 | 14.00 | 14.00 | 14.00 |
| BIC | 64.47 | 64.47 | 64.47 | 64.47 | 64.47 | 64.47 | 64.47 |
| E | 0.80 | 0.74 | 0.74 | 0.64 | 0.78 | 0.80 | 0.74 |

^1^ The number of the iterations the whole algorithm, not the ones of the EM algorithm performed at each step

^2^by rounding

**Table S14: Results of the iterative LCA for the third simulation scenario for the poorly chosen starting values with only positive pairwise dependencies allowed**

| Parameter | Starting values 1 | Starting values 2 | Starting values 3 |
| --- | --- | --- | --- |
| Prevalence  (lCl,uCl) | 0.500  (0.490,0.510) | 0.177  (0.150,0.165) | 0.023  (0.020,0.026) |
| Sen 1  (lCl,uCl) | 0.037  (0.033,0.040) | 0.121  (0.115,0.128) | 0.977  (0.974,0.980) |
| Sen 2  (lCl,uCl) | 0.215  (0.207,0.223) | 0.862  (0.856,0.869) | 0.759  (0.751,0.768) |
| Sen 3  (lCl,uCl) | 0.165  (0.158,0.172) | 0.825  (0.818,0.833) | 0.707  (0.698,0.761) |
| Spe 1  (lCl,uCl) | 0.963  (0.960,0.967) | 0.981  (0.978,0.985) | 0.985  (0.983,0.988) |
| Spe 2  (lCl,uCl) | 0.785  (0.777,0.793) | 0.924  (0.919,0.930) | 0.797  (0.790,0.806) |
| Spe 3  (lCl,uCl) | 0.835  (0.828,0.842) | 0.977  (0.974,0.980) | 0.847  (0.841,0.855) |
| ${}_{12}^{+}$(std.) | 0.013 (0.168) | 0.000 (0.000) | 0.011 (0.169) |
| ${}_{13}^{+}$(std.) | 0.013 (0.185) | 0.000 (0.000) | 0.010 (0.153) |
| ${}_{23}^{+}$(std.) | 0.094 (0.617) | 0.000 (0.000) | 0.106 (0.546) |
| ${}_{123}^{+}$(std.) | 0.007 (0.245) | 0.000^2^ (0.005) | -0.001 (-0.024) |
| ${}_{12}^{-}$ (std.) | 0.013 (0.168) | 0.002 (0.045) | 0.001 (0.018) |
| ${}_{13}^{-}$ (std.) | 0.013 (0.185) | 0.001 (0.061) | 0.001 (0.020) |
| ${}_{23}^{-}$ (std.) | 0.094 (0.617) | 0.003 (0.071) | 0.087 (0.601) |
| ${}_{123}^{-}$(std.) | -0.007 (-0.245) | -0.002 (-0.289) | -0.001 (-0.04) |
| Iterations^1^ | 3 | 6 | 4 |
| LL | -9522.498 | -9522.498 | -9522.498 |
| AIC | 14.00 | 14.00 | 14.00 |
| BIC | 64.47 | 64.47 | 64.47 |
| E | 0.00 | 0.77 | 0.01 |

^1^ The number of the iterations the whole algorithm, not the ones of the EM algorithm performed at each step

^2^by rounding

**Table S15: Results of the Bayesian LCA for the well-chosen starting values in the first simulation scenario**

| Para-meter | Starting values 1 | Starting values 2 | Starting values 3 | Starting values 4 | Starting values 5 | Starting values 6 |
| --- | --- | --- | --- | --- | --- | --- |
| Prev  lCl  uCl | 0.300  0.293  0.307 | 0.301  0.286  0.316 | 0.300  0.285  0.315 | 0.338  0.330  0.346 | 0.369  0.361  0.377 | 0.369  0.361  0.377 |
| Sen 1  lCl  uCl | 0.896  0.887  0.906 | 0.882  0.860  0.904 | 0.882  0.860  0.904 | 0.900  0.810  0.909 | 0.912  0.904  0.920 | 0.912  0.904  0.920 |
| Sen 2  lCl  uCl | 0.845  0.835  0.855 | 0.833  0.811  0.854 | 0.832  0.811  0.853 | 0.886  0.877  0.895 | 0.828  0.818  0.838 | 0.828  0.818  0.838 |
| Sen 3  lCl  uCl | 0.900  0.891  0.909 | 0.899  0.878  0.920 | 0.901  0.879  0.923 | 0.899  0.890  0.908 | 0.936  0.929  0.943 | 0.936  0.929  0.943 |
| Spe 1  lCl  uCl | 0.947  0.941  0.953 | 0.941  0.929  0.953 | 0.940  0.928  0.952 | 0.947  0.941  0.953 | 0.925  0.918  0.932 | 0.925  0.918  0.932 |
| Spe 2  lCl  uCl | 0.950  0.944  0.956 | 0.949  0.938  0.960 | 0.947  0.935  0.959 | 0.953  0.947  0.960 | 0.931  0.924  0.938 | 0.931  0.924  0.938 |
| Spe 3  lCl  uCl | 0.989  0.986  0.992 | 0.985  0.974  0.996 | 0.985  0.973  0.997 | 0.989  0.986  0.992 | 0.981  0.977  0.985 | 0.981  0.977  0.985 |
| ${}_{12}^{+}$ | 0.020 | 0.0223 | 0.021 | 0.017 | 0.019 | 0.018 |
| ${}_{13}^{+}$ | 0.000^1^ | 0.008 | 0.008 | 0.003 | 0.004 | 0.004 |
| ${}_{23}^{+}$ | 0.002 | 0.007 | 0.008 | -0.002 | 0.011 | 0.012 |
| ${}_{123}^{+}$ | 0.000^1^ | 0.000^1^ | 0.000^1^ | 0.000^1^ | 0.019 | 0.000^1^ |
| ${}_{12}^{-}$ | 0.005 | 0.009 | 0.011 | 0.003 | 0.004 | 0.019 |
| ${}_{13}^{-}$ | 0.003 | 0.004 | 0.004 | 0.003 | 0.007 | 0.003 |
| ${}_{23}^{-}$ | 0.004 | 0.005 | 0.005 | 0.003 | -0.001 | 0.007 |
| ${}_{123}^{-}$ | -0.001 | 0.000^1^ | 0.000^1^ | 0.000^1^ | -0.001 | 0.000^1^ |
| Iterations | 10000 | 10000 | 10000 | 10000 | 10000 | 10000 |

^1^by rounding

**Table S16: Results of the Bayesian LCA for the well-chosen starting values in the second simulation scenario**

| Para-meter | Starting values 1 | Starting values 2 | Starting values 3 | Starting values 4 | Starting values 5 | Starting values 6 |
| --- | --- | --- | --- | --- | --- | --- |
| Prev  lCl  uCl | 0.400  0.394  0.406 | 0.402  0.391  0.413 | 0.402  0.392  0.412 | 0.368  0.349  0.387 | 0.423  0.412  0.434 | 0.423  0.412  0.434 |
| Sen 1  lCl  uCl | 0.901  0.893  0.909 | 0.906  0.885  0.927 | 0.908  0.888  0.928 | 0.854  0.831  0.877 | 0.874  0.851  0.897 | 0.872  0.848  0.896 |
| Sen 2  lCl  uCl | 0.701  0.691  0.711 | 0.697  0.680  0.714 | 0.695  0.678  0.716 | 0.760  0.737  0.783 | 0.660  0.642  0.678 | 0.654  0.636  0.672 |
| Sen 3  lCl  uCl | 0.651  0.641  0.661 | 0.646  0.629  0.663 | 0.647  0.629  0.665 | 0.719  0.696  0.742 | 0.632  0.612  0.652 | 0.632  0.613  0.651 |
| Spe 1  lCl  uCl | 0.991  0.988  0.994 | 0.996  0.987  1.000 | 0.997  0.991  1.000 | 0.922  0.897  1.000 | 0.998  0.994  1.000 | 0.997  0.993  1.000 |
| Spe 2  lCl  uCl | 0.800  0.792  0.808 | 0.799  0.785  0.813 | 0.798  0.784  0.812 | 0.813  0.805  0.831 | 0.784  0.770  0.798 | 0.783  0.769  0.797 |
| Spe 3  lCl  uCl | 0.851  0.844  0.858 | 0.849  0.835  0.863 | 0.849  0.835  0.863 | 0.868  0.851  0.885 | 0.854  0.839  0.869 | 0.854  0.839  0.869 |
| ${}_{12}^{+}$ | -0.005 | -0.003 | 0.000^1^ | 0.002 | 0.031 | 0.033 |
| ${}_{13}^{+}$ | -0.006 | -0.002 | -0.003 | -0.012 | 0.008 | 0.008 |
| ${}_{23}^{+}$ | 0.118 | 0.115 | 0.115 | 0.095 | 0.116 | 0.117 |
| ${}_{123}^{+}$ | 0.008 | 0.009 | 0.009 | 0.010 | 0.008 | 0.008 |
| ${}_{12}^{-}$ | 0.003 | 0.001 | 0.000^1^ | 0.005 | 0.000^1^ | 0.000^1^ |
| ${}_{13}^{-}$ | 0.003 | 0.001 | 0.000^1^ | 0.009 | 0.000^1^ | 0.000^1^ |
| ${}_{23}^{-}$ | 0.088 | 0.092 | 0.092 | 0.073 | 0.1011 | 0.101 |
| ${}_{123}^{-}$ | 0.000^1^ | 0.000^1^ | 0.000^1^ | 0.003 | 0.000^1^ | 0.000^1^ |
| Iterations | 10000 | 10000 | 10000 | 10000 | 10000 | 10000 |

^1^by rounding

**Table S17: Results of the Bayesian LCA for the well-chosen starting values in the third simulation scenario**

| Para-meter | Starting values 1 | Starting values 2 | Starting values 3 | Starting values 4 | Starting values 5 | Starting values 6 |
| --- | --- | --- | --- | --- | --- | --- |
| Prev  lCl  uCl | 0.029  0.026  0.032 | 0.028  0.022  0.034 | 0.028  0.023  0.033 | 0.018  0.011  0.025 | 0.022  0.012  0.032 | 0.011  0.008  0.014 |
| Sen 1  lCl  uCl | 0.900  0.891  0.909 | 0.901  0.876  0.926 | 0.901  0.876  0.926 | 0.851  0.826  0.876 | 0.930  0.922  0.938 | 0.792  0.765  0.819 |
| Sen 2  lCl  uCl | 0.700  0.689  0.711 | 0.697  0.672  0.752 | 0.698  0.673  0.723 | 0.752  0.727  0.777 | 0.659  0.647  0.671 | 0.800  0.775  0.825 |
| Sen 3  lCl  uCl | 0.649  0.637  0.661 | 0.647  0.622  0.673 | 0.646  0.621  0.672 | 0.722  0.697  0.747 | 0.619  0.607  0.631 | 0.800  0.776  0.826 |
| Spe 1  lCl  uCl | 0.989  0.897  0.991 | 0.988  0.983  0.993 | 0.988  0.983  0.993 | 0.979  0.973  0.985 | 0.983  0.974  0.982 | 0.967  0.964  0.970 |
| Spe 2  lCl  uCl | 0.800  0.796  0.804 | 0.799  0.794  0.804 | 0.799  0.794  0.804 | 0.797  0.792  0.802 | 0.788  0.783  0.793 | 0.789  0.785  0.793 |
| Spe 3  lCl  uCl | 0.850  0.846  0.854 | 0.849  0.845  0.853 | 0.849  0.845  0.853 | 0.847  0.842  0.852 | 0.840  0.835  0.845 | 0.840  0.836  0.844 |
| ${}_{12}^{+}$ | 0.016 | 0.014 | 0.015 | 0.039 | 0.011 | 0.053 |
| ${}_{13}^{+}$ | 0.008 | 0.009 | 0.006 | 0.031 | 0.006 | 0.053 |
| ${}_{23}^{+}$ | 0.129 | 0.128 | 0.127 | 0.085 | 0.126 | 0.056 |
| ${}_{123}^{+}$ | 0.010 | 0.009 | 0.009 | 0.000^1^ | 0.006 | -0.008 |
| ${}_{12}^{-}$ | 0.000^1^ | 0.001 | 0.000^1^ | 0.004 | 0.004 | 0.009 |
| ${}_{13}^{-}$ | 0.000^1^ | 0.001 | 0.000^1^ | 0.004 | 0.004 | 0.009 |
| ${}_{23}^{-}$ | 0.086 | 0.086 | 0.086 | 0.087 | 0.091 | 0.091 |
| ${}_{123}^{-}$ | 0.000^1^ | 0.000^1^ | 0.000^1^ | -0.002 | -0.002 | -0.005 |
| Iterations | 10000 | 10000 | 10000 | 10000 | 10000 | 10000 |

^1^by rounding

**Table S18: Results of the Bayesian LCA for the well-chosen starting values in the forth simulation scenario**

| Para-meter | Starting values 1 | Starting values 2 | Starting values 3 | Starting values 4 | Starting values 5 | Starting values 6 |
| --- | --- | --- | --- | --- | --- | --- |
| Prev  lCl  uCl | 0.400  0.393  0.407 | 0.400  0.379  0.421 | 0.397  0.371  0.423 | 0.393  0.383  0.403 | 0.365  0.325  0.405 | 0.315  0.295  0.335 |
| Sen 1  lCl  uCl | 0.800  0.790  0.810 | 0.807  0.763  0.851 | 0.815  0.771  0.859 | 0.853  0.839  0.876 | 0.613  0.574  0.652 | 0.787  0.738  0.836 |
| Sen 2  lCl  uCl | 0.660  0.650  0.670 | 0.673  0.636  0.710 | 0.670  0.637  0.703 | 0.716  0.699  0.733 | 0.625  0.582  0.668 | 0.778  0.728  0.838 |
| Sen 3  lCl  uCl | 0.701  0.691  0.711 | 0.717  0.678  0.756 | 0.719  0.683  0.755 | 0.753  0.742  0.764 | 0.615  0.576  0.654 | 0.781  0.732  0.830 |
| Spe 1  lCl  uCl | 0.950  0.944  0.956 | 0.955  0.926  0.984 | 0.956  0.924  0.988 | 0.978  0.967  0.989 | 0.802  0.773  0.831 | 0.849  0.824  0.874 |
| Spe2  lCl  uCl | 0.850  0.842  0.858 | 0.859  0.832  0.886 | 0.855  0.826  0.029 | 0.882  0.870  0.894 | 0.803  0.769  0.837 | 0.840  0.812  0.868 |
| Spe 3  lCl  uCl | 0.881  0.873  0.889 | 0.892  0.866  0.918 | 0.890  0.858  0.922 | 0.911  0.899  0.923 | 0.801  0.771  0.831 | 0.844  0.817  0.871 |
| ${}_{12}^{+}$ | 0.036 | 0.016 | 0.026 | -0.016 | 0.079 | 0.037 |
| ${}_{13}^{+}$ | 0.044 | 0.032 | 0.030 | -0.009 | 0.138 | 0.054 |
| ${}_{23}^{+}$ | 0.070 | 0.074 | 0.075 | 0.069 | 0.112 | 0.054 |
| ${}_{123}^{+}$ | 0.005 | 0.007 | 0.005 | 0.005 | -0.009 | -0.006 |
| ${}_{12}^{-}$ | 0.017 | 0.021 | 0.015 | 0.006 | 0.070 | 0.030 |
| ${}_{13}^{-}$ | 0.018 | 0.016 | 0.015 | 0.009 | 0.066 | 0.045 |
| ${}_{23}^{-}$ | 0.057 | 0.044 | 0.045 | 0.019 | 0.080 | 0.049 |
| ${}_{123}^{-}$ | -0.004 | -0.004 | -0.001 | -0.002 | -0.016 | -0.004 |
| Iterations | 10000 | 10000 | 10000 | 10000 | 10000 | 10000 |

**Table S19: Results of the Bayesian LCA for the well-chosen starting values in the fivth simulation scenario**

| Para-meter | Starting values 1 | Starting values 2 | Starting values 3 | Starting values 4 | Starting values 5 | Starting values 6 |
| --- | --- | --- | --- | --- | --- | --- |
| Prev  lCl  uCl | 0.200  0.196  0.204 | 0.201  0.180  0.222 | -^2^ | 0.157  0.161  0.163 | 0.180  0.166  0.194 | 0.221  0.212  0.230 |
| Sen 1  lCl  uCl | 0.717  0.706  0.728 | 0.700  0.665  0.735 | -^2^ | 0.772  0.761  0.783 | 0.685  0.642  0.728 | 0.679  0.659  0.699 |
| Sen 2  lCl  uCl | 0.651  0.641  0.661 | 0.637  0.602  0.672 | -^2^ | 0.709  0.694  0.722 | 0.639  0.592  0.686 | 0.623  0.595  0.651 |
| Sen 3  lCl  uCl | 0.970  0.965  0.975 | 0.959  0.928  0.990 | -^2^ | 0.976  0.946  0.996 | 0.955  0.937  0.973 | 0.940  0.917  0.963 |
| Spe 1  lCl  uCl | 0.979  0.976  0.982 | 0.976  0.961  0.991 | -^2^ | 0.958  0.950  0.966 | 0.955  0.944  0.966 | 0.986  0.978  0.994 |
| Spe 2  lCl  uCl | 0.991  0.988  0.994 | 0.987  0.974  1.000 | -^2^ | 0.972  0.964  0.980 | 0.972  0.965  0.979 | 0.998  0.995  1.000 |
| Spe 3  lCl  uCl | 0.980  0.976  0.984 | 0.978  0.956  1.000 | -^2^ | 0.936  0.925  0.947 | 0.954  0.940  0.968 | 0.996  0.988  1.000 |
| ${}_{12}^{+}$ | 0.127 | 0.131 | -^2^ | 0.124 | 0.155 | 0.126 |
| ${}_{13}^{+}$ | 0.010 | 0.009 | -^2^ | 0.009 | 0.012 | 0.013 |
| ${}_{23}^{+}$ | 0.003 | 0.008 | -^2^ | 0.008 | 0.008 | 0.006 |
| ${}_{123}^{+}$ | 0.004 | 0.007 | -^2^ | 0.000^1^ | 0.006 | 0.013 |
| ${}_{12}^{-}$ | 0.002 | 0.006 | -^2^ | 0.013 | 0.016 | 0.000^1^ |
| ${}_{13}^{-}$ | 0.003 | 0.009 | -^2^ | 0.022 | 0.027 | 0.001 |
| ${}_{23}^{-}$ | 0.003 | 0.007 | -^2^ | 0.018 | 0.022 | 0.000^1^ |
| ${}_{123}^{-}$ | 0.000^1^ | -0.004 | -^2^ | -0.010 | -0.013 | 0.000^1^ |
| Iterations | 10000 | 10000 | 8000^3^ | 10000 | 10000 | 10000 |

^1^by rounding

^2^model did not converge and the algorithm aborted the calculation after 8000 Iterations

**Table S20: Results of the Bayesian LCA for the poorly chosen starting values in the first simulation scenario**

| Parameter | Starting values 1 | Starting values 2 | Starting values 3 |
| --- | --- | --- | --- |
| Prevalence  (lCl,uCl) | 0.791  (0.501, 1.000) | 0.262  (0.250, 0.274) | 0.263  (0.242, 0.284) |
| Sen 1  (lCl,uCl) | 0.335  (0.186, 0.484) | 0.851  (0.836, 0.866) | 0.716  (0.690, 0.742) |
| Sen 2  (lCl,uCl) | 0.329  (0.164, 0.658) | 0.853  (0.838, 0.868) | 0.700  (0.676, 0.724) |
| Sen 3  (lCl,uCl) | 0.317  (0.163, 0.471) | 0.850  (0.835, 0.865) | 0.821  (0.747, 0.895) |
| Spe 1  (lCl,uCl) | 0.522  (0.257, 0.787) | 0.900  (0.889, 0.911) | 0.826  (0.804, 0.848) |
| Spe 2  (lCl,uCl) | 0.545  (0.277, 0.813) | 0.933  (0.919, 0.947) | 0.843  (0.813, 0.873) |
| Spe 3  (lCl,uCl) | 0.555  (0.285, 0.825) | 0.932  (0.921,0.943) | 0.897  (0.855, 0.939) |
| ${}_{12}^{+}$ | 0.132 | 0.040 | 0.085 |
| ${}_{13}^{+}$ | 0.139 | 0.044 | 0.092 |
| ${}_{23}^{+}$ | 0.133 | 0.037 | 0.077 |
| ${}_{123}^{+}$ | 0.048 | -0.007 | -0.028 |
| ${}_{12}^{-}$ | 0.036 | 0.022 | 0.090 |
| ${}_{13}^{-}$ | 0.038 | 0.036 | 0.071 |
| ${}_{23}^{-}$ | 0.035 | 0.021 | 0.069 |
| ${}_{123}^{-}$ | -0.008 | -0.006 | -0.041 |
| Iterations | 10000 | 10000 | 10000 |

**Table S21: Results of the Bayesian LCA for the poorly chosen starting values in the second simulation scenario**

| Parameter | Starting values 1 | Starting values 2 | Starting values 3 |
| --- | --- | --- | --- |
| Prevalence  (lCl,uCl) | 0.290  (0.155, 0.425) | 0.333  (0.315, 0.351) | 0.975  (0.970, 0.980) |
| Sen 1  (lCl,uCl) | 0.595  (0.384, 0.806) | 0.882  (0.855, 0.909) | 0.390  (0.383, 0.397) |
| Sen 2  (lCl,uCl) | 0.764  (0.533, 0.995) | 0.813  (0.790, 0.836) | 0.427  (0.420, 0.434) |
| Sen 3  (lCl,uCl) | 0.752  (0.537, 0.967) | 0.787  (0.763, 0.811) | 0.378  (0.370, 0.386) |
| Spe 1  (lCl,uCl) | 0.740  (0.656, 0.824) | 0.893  (0.876, 0.910) | 0.856  (0.832, 0.880) |
| Spe 2  (lCl,uCl) | 0.766  (0.651, 0.881) | 0.810  (0.793, 0.829) | 0.928  (0.904, 0.952) |
| Spe 3  (lCl,uCl) | 0.819  (0.701, 0.937) | 0.870  (0.855,0.885) | 0.984  (0.956, 1.000) |
| ${}_{12}^{+}$ | 0.012 | 0.013 | 0.109 |
| ${}_{13}^{+}$ | 0.014 | -0.005 | 0.110 |
| ${}_{23}^{+}$ | 0.027 | 0.048 | 0.164 |
| ${}_{123}^{+}$ | 0.000^1^ | 0.004 | 0.014 |
| ${}_{12}^{-}$ | 0.067 | -0.006 | 0.025 |
| ${}_{13}^{-}$ | 0.062 | -0.005 | 0.006 |
| ${}_{23}^{-}$ | 0.089 | 0.079 | 0.005 |
| ${}_{123}^{-}$ | -0.017 | 0.005 | -0.001 |
| Iterations | 10000 | 10000 | 10000 |

^1^by rounding

**Table S22: Results of the Bayesian LCA for the poorly chosen starting values in the third scenario**

| Parameter | Starting values 1 | Starting values 2 | Starting values 3 |
| --- | --- | --- | --- |
| Prevalence  (lCl,uCl) | 0.894  (0.815, 0.973) | 0.036  (0.033,0.039) | 0.027  (0.021, 0.033) |
| Sen 1  (lCl,uCl) | 0.018  (0.003, 0.033) | 0.796  (0.725, 0.867) | 0.603  (0.575, 0.631) |
| Sen 2  (lCl,uCl) | 0.181  (0.149, 0.213) | 0.759  (0.705, 0.813) | 0.898  (0.873, 0.923) |
| Sen 3  (lCl,uCl) | 0.140  (0.111, 0.169) | 0.725  (0.670, 0.780) | 0.845  (0.810, 0.880) |
| Spe 1  (lCl,uCl) | 0.689  (0.451, 0.927) | 0.990  (0.987, 0.993) | 0.979  (0.976, 0.982) |
| Spe 2  (lCl,uCl) | 0.413  (0.160, 0.666) | 0.804  (0.799, 0.809) | 0.806  (0.800, 0.812) |
| Spe 3  (lCl,uCl) | 0.522  (0.262, 0.782) | 0.854  (0.850, 0.858) | 0.855  (0.849, 0.861) |
| ${}_{12}^{+}$ | 0.005 | -0.003 | 0.008 |
| ${}_{13}^{+}$ | 0.005 | -0.019 | 0.021 |
| ${}_{23}^{+}$ | 0.082 | 0.094 | 0.033 |
| ${}_{123}^{+}$ | 0.002 | 0.012 | 0.000^1^ |
| ${}_{12}^{-}$ | 0.010 | 0.000^1^ | 0.002 |
| ${}_{13}^{-}$ | 0.031 | 0.000^1^ | 0.002 |
| ${}_{23}^{-}$ | 0.073 | 0.083 | 0.082 |
| ${}_{123}^{-}$ | -0.021 | 0.000^1^ | 0.000^1^ |
| Iterations | 10000 | 10000 | 10000 |

^1^by rounding

**Table S23: Results of the Bayesian LCA for the poorly chosen starting values in the forth simulation scenario**

| Parameter | Starting values 1 | Starting values 2 | Starting values 3 |
| --- | --- | --- | --- |
| Prevalence  (lCl,uCl) | 0.418  (0.130, 0.706) | 0.403  (0.385, 0.421) | 0.373  (0.364, 0.382) |
| Sen 1  (lCl,uCl) | 0.444  (0.246, 0.642) | 0.635  (0.611, 0.659) | 0.775  (0.753, 0.797) |
| Sen 2  (lCl,uCl) | 0.402  (0.239, 0.565) | 0.639  (0.617, 661) | 0.782  (0.760, 0.804) |
| Sen 3  (lCl,uCl) | 0.349  (0.159, 0.539) | 0.764  (0.737, 0.791) | 0.858  (0.837, 0.879) |
| Spe 1  (lCl,uCl) | 0.634  (0.518, 0.750) | 0.847  (0.828, 0.866) | 0.901  (0.889, 0.913) |
| Spe 2  (lCl,uCl) | 0.612  (0.489, 0.735) | 0.843  (0.824, 0.862) | 0.899  (0.885, 0.913) |
| Spe 3  (lCl,uCl) | 0.629  (0.527, 731) | 0.927  (0.910, 0.944) | 0.948  (0.933, 0.963) |
| ${}_{12}^{+}$ | 0.064 | 0.083 | 0.017 |
| ${}_{13}^{+}$ | 0.081 | 0.077 | 0.004 |
| ${}_{23}^{+}$ | 0.079 | 0.076 | 0.000^1^ |
| ${}_{123}^{+}$ | 0.016 | 0.015 | 0.000^1^ |
| ${}_{12}^{-}$ | 0.088 | 0.044 | 0.004 |
| ${}_{13}^{-}$ | 0.107 | 0.036 | 0.007 |
| ${}_{23}^{-}$ | 0.097 | 0.037 | 0.009 |
| ${}_{123}^{-}$ | -0.022 | -0.011 | 0.000^1^ |
| Iterations | 10000 | 10000 | 10000 |

^1^by rounding

**Table S24: Results of the Bayesian LCA for the poorly chosen starting values in the fivth simulation scenario**

| Parameter | Starting values 1 | Starting values 2 | Starting values 3 |
| --- | --- | --- | --- |
| Prevalence  (lCl,uCl) | 0.035  (0.007, 0.063) | 0.193  (0.183, 0.203) | 0.158  (0.154, 0.162) |
| Sen 1  (lCl,uCl) | 0.406  (0.167, 0.645) | 0.801  (0.776 ,0.826) | 0.906  (0.885, 0.927) |
| Sen 2  (lCl,uCl) | 0.351  (0.074, 0.628) | 0.724  (0.691, 0.757) | 0.825  (0.806, 0.844) |
| Sen 3  (lCl,uCl) | 0.556  (0.313, 0.799) | 0.771  (0.752, 0.790) | 0.970  (0.745, 0.795) |
| Spe 1  (lCl,uCl) | 0.847  (0.838, 0.856) | 0.990  (0.980, 1.000) | 0.979  (0.975, 0.983) |
| Spe 2  (lCl,uCl) | 0.865  (0.855,0.875) | 0.998  (0.995, 1.000) | 0.990  (0.985, 0.995) |
| Spe 3  (lCl,uCl) | 0.802  (0.787, 0.817) | 0.922  (0.912, 0.932) | 0.931  (0.926, 0.936) |
| ${}_{12}^{+}$ | -0.004 | 0.055 | 0.009 |
| ${}_{13}^{+}$ | 0.023 | 0.102 | 0.012 |
| ${}_{23}^{+}$ | 0.010 | 0.126 | 0.009 |
| ${}_{123}^{+}$ | 0.002 | -0.037 | 0.000^1^ |
| ${}_{12}^{-}$ | 0.101 | 0.001 | 0.002 |
| ${}_{13}^{-}$ | 0.110 | 0.008 | 0.004 |
| ${}_{23}^{-}$ | 0.103 | 0.000^1^ | 0.003 |
| ${}_{123}^{-}$ | -0.066 | 0.000^1^ | 0.000^1^ |
| Iterations | 10000 | 10000 | 10000 |

^1^by rounding

**Table S25: Results of the Bayesian LCA for the well-chosen starting values in the third simulation scenario with only positive pairwise dependencies allowed**

| Para-meter | Starting values 1 | Starting values 2 | Starting values 3 | Starting values 4 | Starting values 5 | Starting values 6 |
| --- | --- | --- | --- | --- | --- | --- |
| Prev  lCl  uCl | 0.028  0.026  0.030 | 0.024  0.019  0.029 | 0.023  0.018  0.028 | 0.017  0.012  0.022 | 0.019  0.011  0.027 | 0.011  0.008  0.014 |
| Sen 1  lCl  uCl | 0.901  0.892  0.910 | 0.902  0.877  0.927 | 0.903  0.878  0.928 | 0.851  0.825  0.877 | 0.930  0.922  0.938 | 0.791  0.765  0.817 |
| Sen 2  lCl  uCl | 0.698  0.686  0.710 | 0.697  0.672  0.722 | 0.696  0.671  0.721 | 0.751  0.726  0.776 | 0.659  0.647  0.671 | 0.800  0.775  0.825 |
| Sen 3  lCl  uCl | 0.648  0.636  0.660 | 0.648  0.623  0.673 | 0.648  0.623  0.673 | 0.721  0.696  0.746 | 0.619  0.607  0.631 | 0.801  0.776  0.826 |
| Spe 1  lCl  uCl | 0.988  0.986  0.990 | 0.984  0.980  0.988 | 0.984  0.979  0.989 | 0.978  0.973  0.983 | 0.980  0.973  0.987 | 0.967  0.964  0.970 |
| Spe 2  lCl  uCl | 0.799  0.795  0.803 | 0.797  0.793  0.801 | 0.797  0.792  0.802 | 0.796  0.791  0.801 | 0.787  0.782  0.792 | 0.789  0.785  0.793 |
| Spe 3  lCl  uCl | 0.849  0.845  0.853 | 0.847  0.843  0.850 | 0.847  0.843  0.850 | 0.846  0.841  0.851 | 0.839  0.834  0.844 | 0.840  0.836  0.844 |
| ${}_{12}^{+}$ | 0.027 | 0.031 | 0.031 | 0.054 | 0.022 | 0.073 |
| ${}_{13}^{+}$ | 0.026 | 0.029 | 0.029 | 0.053 | 0.021 | 0.075 |
| ${}_{23}^{+}$ | 0.139 | 0.132 | 0.136 | 0.100 | 0.136 | 0.070 |
| ${}_{123}^{+}$ | 0.004 | 0.001 | 0.000^1^ | -0.010 | 0.002 | -0.019 |
| ${}_{12}^{-}$ | 0.001 | 0.003 | 0.003 | 0.005 | 0.005 | 0.009 |
| ${}_{13}^{-}$ | 0.001 | 0.003 | 0.003 | 0.004 | 0.005 | 0.009 |
| ${}_{23}^{-}$ | 0.086 | 0.087 | 0.087 | 0.088 | 0.092 | 0.091 |
| ${}_{123}^{-}$ | 0.000^1^ | 0.000^1^ | 0.000^1^ | -0.002 | -0.003 | -0.004 |
| Iterations | 10000 | 10000 | 10000 | 10000 | 10000 | 10000 |

^1^by rounding

**Table S26: Results of the Bayesian LCA for the poorly chosen starting values in the third simulation scenario with only positive pairwise dependencies allowed**

| Parameter | Starting values 1 | Starting values 2 | Starting values 3 |
| --- | --- | --- | --- |
| Prevalence  (lCl,uCl) | 0.711  (0.372, 1.000) | 0.034  (0.031, 0.037) | 0.024  (0.019, 0.029) |
| Sen 1  (lCl,uCl) | 0.058  (0.000, 0.181) | 0.811  (0.716, 0.906) | 0.600  (0.573, 0.627) |
| Sen 2  (lCl,uCl) | 0.219  (0.057, 0.381) | 0.721  (0.660, 0.782) | 0.897  (0.872, 0.922) |
| Sen 3  (lCl,uCl) | 0.185  (0.021, 0.349) | 0.686  (0.622, 0.75) | 0.846  (0.811, 0.881) |
| Spe 1  (lCl,uCl) | 0.754  (0.483, 1.000) | 0.988  (0.984, 0.992) | 0.977  (0.974, 0.980) |
| Spe 2  (lCl,uCl) | 0.552  (0.334, 0.770) | 0.801  (0.796, 0.806) | 0.804  (0.799, 0.809) |
| Spe 3  (lCl,uCl) | 0.615  (0.386, 0.844) | 0.851  (0.847, 0.855) | 0.853  (0.848, 0.858) |
| ${}_{12}^{+}$ | 0.009 | 0.026 | 0.029 |
| ${}_{13}^{+}$ | 0.010 | 0.021 | 0.044 |
| ${}_{23}^{+}$ | 0.073 | 0.119 | 0.044 |
| ${}_{123}^{+}$ | 0.003 | 0.007 | -0.011 |
| ${}_{12}^{-}$ | 0.028 | 0.000^1^ | 0.003 |
| ${}_{13}^{-}$ | 0.030 | 0.000^1^ | 0.003 |
| ${}_{23}^{-}$ | 0.101 | 0.085 | 0.083 |
| ${}_{123}^{-}$ | -0.007 | 0.000^1^ | 0.000^1^ |
| Iterations | 10000 | 10000 | 10000 |

^1^by rounding
